# Tobacco intercropping enhances soil fertility by improving synergic interactions between soil physicochemical and microbial properties

**DOI:** 10.3389/fmicb.2025.1647493

**Published:** 2025-09-02

**Authors:** Kaiyuan Gu, Xianglu Liu, Ming Liu, Xu Wei, Juan Li, Yanxia Hu, Yonglei Jiang, Yi Chen, Dexun Wang, Yanming Yang, Jiaen Su, Longchang Wang

**Affiliations:** ^1^College of Agronomy and Biotechnology, Southwest University/Engineering Research Center of South Upland Agriculture, Ministry of Education, Chongqing, China; ^2^Yunnan Tobacco Company, Dali State Branch, Dali, China; ^3^Yunnan Academy of Tobacco Agricultural Sciences, Kunming, China

**Keywords:** soil microbiome, microbial network complexity, soil ecosystem resilience, nitrogen-fixing symbiosis, soil nutrient cycling

## Abstract

**Introduction:**

Intercropping tobacco with other crops has been shown to upregulate soil health by fostering synergistic interactions between physicochemical and microbial properties. This study aims to evaluate the impact of intercropping on physicochemical attributes, rhizospheric microbial community, and functional dynamics of soil cultivated with tobacco plants.

**Methods:**

A field experiment was comprised with five treatments, such as tobacco monoculture (TT), soybean monoculture (SS), maize monoculture (MM), tobacco–soybean intercropping (TS), and tobacco–maize intercropping (TM). Soil nutrients observed, while bacterial and fungal community profiles were assessed through high-throughput sequencing targeting the 16S rDNA and ITS hypervariable regions. Microbial interactions and network resilience were assessed through co-occurrence network analysis.

**Results:**

Intercropping significantly improved the soil nutrient properties. Compared with tobacco monoculture (TT), the tobacco–soybean intercropping (TS) treatment enhanced cation exchange capacity (CEC), total nitrogen (TN), available phosphorus (AP), and available potassium (AK) by 13.9, 13.9, 43.8, and 129.1%, respectively. Tobacco–maize intercropping (TM) enhanced CEC (26.7%) and AK (9.7%). Both intercropping models significantly increased bacterial species richness in tobacco soil, whereas fungal diversity was more pronounced under monoculture conditions. Intercropping favored the proliferation of *Proteobacteria* and *Basidiomycota,* while concurrently suppressing *Ascomycota*. Tobacco–maize intercropping specifically augmented nitrifying bacteria and *Actinobacteria*, while tobacco–soybean intercropping predominantly facilitated the recruitment of symbiotic fungi. Intercropping intensified microbial network complexity and modularity, upregulate ecosystem resilience to disturbances. Mantel analysis indicated that the bacterial community structure was primarily influenced by soil pH, whereas fungal communities exhibited strong combinations with available potassium and phosphorus.

**Discussion:**

Intercropping systems substantially improved soil ecological functionality by modulating microbial community composition and nutrient dynamics. Tobacco–maize intercropping reinforced soil ecosystem stability through enrichment of functional microorganisms and optimization of community architecture, while tobacco–soybean intercropping leveraged nitrogen fixation by legumes to augment nitrogen availability and facilitate the establishment of nitrogen-cycling microbes, demonstrating superior efficacy in enhancing soil fertility. These findings suggest that tobacco intercropping can be sustainable agricultural strategy to maintain soil health and productivity in the era of climate change.

## 1 Introduction

Tobacco (*Nicotiana tabacum* L.), a pivotal model crop in agricultural research and a key economic leaf crop in the southwestern region of China, faces significant challenges under long-term conventional monoculture. Prolonged tobacco monoculture has exposed various agronomic issues, i.e., continuous cropping barriers, nutrient imbalance, microbial community deterioration, and heightened susceptibility to soil-borne diseases. Continuous cropping exacerbates soil structure degradation, enhances the depletion of essential nutrients, such as nitrogen, phosphorus, and potassium, and promotes soil acidification or salinization (Pan et al., 2023). Furthermore, extended monoculture simplifies the soil microbial community, reduces the presence of beneficial microorganisms, and facilitates pathogen accumulation, thereby elevating the risk of disease outbreaks (Lai et al., 2017). Consequently, developing alternative cropping systems that respect soil health and improve crop resilience has become crucial in tobacco cultivation management.

Given the constraints of limited arable land and the growing imperative for sustainable agricultural practices, intercropping has emerged as a viable strategy to mitigate monoculture-related challenges. By staggering the peak demand for light, water, and nutrients among different crops, intercropping confers multiple ecological advantages, including enhanced soil biodiversity, improved resource use efficiency (RUE), reduced pest and weed pressure, and stabilized crop yields (Boudreau, 2013; Afrin et al., 2017; Raseduzzaman and Jensen, 2017). The integrative planting approach, intercropping utilizes spatially optimized crop arrangements to facilitate complementary resource utilization, maximizing system productivity (Yin et al., 2020; Raza et al., 2021). For example, the inclusion of legumes with cereals in intercropping systems enhances atmospheric nitrogen fixation, contributing additional nitrogen to the system, and improving the availability of other nutrients, such as phosphorus (Li et al., 2003; Duchene et al., 2017; Xia et al., 2019). Moreover, intercropping significantly improves the soil physicochemical and biological attributes, including augmenting organic matter content (OMC), increasing soil permeability, and fostering rhizosphere microbial diversity (Te et al., 2022; Peng et al., 2024; Wang A. et al., 2024; Wang G. et al., 2024). Empirical evidence from three-year intercropping study associated with wheat, maize, and faba bean demonstrated that alterations in rhizosphere physicochemical properties, like nutrient availability and pH modulated the bacterial community composition, contingent on the specific crop combinations employed (Song et al., 2007). These observations indicated that the intercropping consistently enhances soil quality and bolsters ecosystem stability. The cultivation approach offering substantial ecological benefits, intercropping presents a promising avenue for advancing soil quality management and optimizing agroecosystem performance. Therefore, elucidating how intercropping influences soil microbial communities and ecosystem functions remains a priority in agronomic research.

In recent years, intercropping has emerged as a sustainable approach to tobacco production, garnering significant attention among researchers. Tobacco intercropping has been shown to increase plant biomass and enhance chemical quality, as well as improve rhizospheric nutrient availability and enzyme activity (Zhou et al., 2023). Intercropping systems involving tobacco and maize have demonstrated significant effects on soil nutrient levels and enzyme activities by regulating the rhizosphere microenvironment (Ma et al., 2024; Yang K. et al., 2025; Yang H. et al., 2025). This approach enhances crop biomass and nutrient accumulation, promotes the growth of beneficial microorganisms, and inhibits the proliferation of harmful microbes. Moreover, previous studies have shown that intercropping with soybean significantly improves the nutrient status of flue-cured tobacco rhizosphere soil and enhances the richness and diversity of microbial communities (Niu et al., 2025). However, current research on the dynamic changes and response mechanisms of soil microbial communities under tobacco intercropping systems remains limited, particularly with respect to microbial community stability and the regulatory mechanisms of co-occurrence networks (Su X. et al., 2024; Su D. et al., 2024; Yang K. et al., 2025; Yang H. et al., 2025). It is worth noting that microbial communities play a crucial role in the transformation of key nutrients such as nitrogen, phosphorus, and potassium. Their community structure, abundance, and functional activity are often closely linked to soil nutrient levels. Previous studies have shown that the abundance of nitrogen-fixing, phosphate-solubilizing, and potassium-solubilizing bacteria is significantly positively correlated with the concentrations of available nitrogen, phosphorus, and potassium in the soil (Richardson et al., 2009; Basak and Biswas, 2009), directly affecting crop nutrient uptake efficiency and soil enzyme activities (Geisseler and Scow, 2014). Xiao et al. (2023) found that intercropping systems enhance soil enzyme activity and microbial biomass by increasing microbial diversity and functional ecological networks, thereby promoting the transformation and accumulation of essential nutrients such as nitrogen, phosphorus, and potassium. Therefore, exploring the relationship between soil microbes and soil nutrients can help to better understand the mechanisms by which tobacco intercropping improves soil ecological functions. This study aims to systematically investigate how tobacco intercropping enhances soil ecological functions by increasing the complexity and stability of microbial networks. By focusing on the integrated effects of tobacco intercropping on soil physicochemical properties and microbial community structure, the study seeks to elucidate the underlying mechanisms through which intercropping improves soil quality, maintains ecological health, and enhances crop productivity, thereby providing a theoretical basis for the optimization of sustainable planting systems.

## 2 Materials and methods

### 2.1 Experimental design

The field experiment was conducted (2023) in Midu County, Dali Bai Autonomous Prefecture, Yunnan Province (25.38 °N, 100.41 °E), China. It observed average annual temperature (17.3 °C) and average annual precipitation (824.4 mm). Before field demonstrations, soil physicochemical properties were observed as available nitrogen (159.97 mg/kg), available phosphorus (84.13 mg/kg), available potassium (673.08 mg/kg), total nitrogen (1.68 g/kg), total phosphorus (1.10 g/kg), total potassium (18.33 g/kg), organic matter (28.15 g/kg), and soil pH (7.87), respectively. Five treatments were established, such as tobacco monoculture (TT), soybean monoculture (SS), maize monoculture (MM), tobacco–soybean intercropping (TS), and tobacco–maize intercropping (TM).

The experiment employed a randomized block design (RBD) with five replicates per treatment, and 24.8 m^2^ plot area for each treatment. For detailed information regarding the field layout and design shown in Supplementary Note S1, Supplementary Figures S1, S2. Tobacco and companion crops were arranged in staggered configurations in the intercropping treatments. Fertilization for tobacco was uniformly applied across all plots, while intercropped species received fertilization based on conventional local practices. The flue-cured tobacco in all five treatments was harvested simultaneously in the month of September, while the intercropped crops were harvested at their respective physiological maturity. To eliminate the effects of crop rotation, all experimental plots were left fallow during the winter after the harvest of tobacco and intercropped crops.

### 2.2 Soil sample collection

After flue-cured tobacco and intercropped crops were harvested on September 27, 2024, soil samples were collected from the topsoil layer (0–20 cm) in each plot using the five-point sampling method. The soil samples from the same plot were combined to create composite samples, which were passed through 2.0 mm sieve for homogenization. Each composite sample was obtained by mixing 5 subsamples within a single replicate plot. A total of 5 replicate plots were established for each treatment, and 5 independent composite samples were collected accordingly. All subsequent analyses were conducted using the plot as the statistical unit. Notably, the sampling points for the TT treatment were located between any two adjacent tobacco plants, for the SS treatment, between any two adjacent soybean plants, and MM treatment, between any two adjacent maize plants. The TS and TM treatments, the sampling points were located between the tobacco strip and the strip of the intercropped crop (soybean or maize). A portion of the soil samples was rapidly stored at −80 °C for DNA extraction, while the remaining part was air-dried for physicochemical analysis.

### 2.3 Determination of soil physicochemical properties

Air-dried soil samples were utilized for the analysis of physicochemical properties. Total nitrogen (TN) was quantified using the semi-micro Kjeldahl method with an automated Kjeldahl analyzer (Kjeltec^™^ 2300, FOSS). Total phosphorus (TP) was measured by the sodium hydroxide fusion–molybdenum antimony colorimetric technique. Total potassium (TK) and hydrolyzable nitrogen (HN) were assessed by sodium hydroxide fusion and flame photometry and alkaline hydrolysis diffusion methods. Available phosphorus (AP) and available potassium (AK) were extracted using sodium bicarbonate and quantified through the molybdenum antimony colorimetric and ammonium acetate extraction and measured by flame photometry. Soil organic matter (SOM) was analyzed by potassium dichromate oxidation method and soil pH with a handy pH meter (potentiometric method) at soil-to-water ratio of 1:2.5. Cation exchange capacity (CEC) was calculated following the extraction of exchangeable Ca^2+^, Mg^2+^, K^+^, and Na^+^ using ammonium acetate.

### 2.4 Soil DNA extraction and high-throughput sequencing

Total microbial DNA was extracted from soil samples using the E. Z. N. A.^®^ Soil DNA Kit (Omega, United States). DNA integrity was verified by agarose gel electrophoresis (1%), and the concentration and purity were assessed using a NanoDrop 2000 spectrophotometer (Thermo Scientific, United States). The bacterial 16S rRNA gene (V3–V4 region) was amplified using forward primer 341F (5′-CCTAYGGGRBGCASCAG-3′) and reverse primer 806R (5′-GGACTACHVGGGTWTCTAAT-3′). The fungal ITS V1 region was amplified using forward primer 1737F (5′-GGAAGTAAAAGTCGTAACAAGG-3′) and reverse primer 2043R (5′-GCTGCGTTCTTCATCGATGC-3′). The PCR reaction mixture contained 5 μL of 5 × buffer, 5 μL of GC buffer (5×), 0.25 μL of FastPfu DNA polymerase (5 U/μL), 2 μL of dNTPs (2.5 mM), 1 μL of each primer (10 μM), DNA template (1 μL), and ddH_2_O (9.75 μL). The amplification protocol consisted of initial denaturation at 98 °C (5 min), annealing at 52 °C for 30 s, extension at 72 °C for 1 min, and final extension at 72 °C for 5 min. PCR products were separated on agarose gels (2%), purified using the AxyPrep DNA Gel Extraction Kit, eluted with Tris–HCl buffer, and confirmed by agarose gel electrophoresis (2%). The concentrations of bacterial and fungal amplicons were determined using the QuantiFluor™-ST system. Sequencing was performed using paired-end (2 × 300 bp) technology on an Illumina Illumina NovaSeq 6000 platform following the standard protocols of Majorbio Bio-Pharm Technology Co., Ltd., Shanghai, China. Raw sequencing data were deposited in the NCBI Sequence Read Archive (SRA, https://submit.ncbi.nlm.nih.gov/subs/sra/) with accession No. PRJNA1251217 and No. PRJNA1251580.

#### 2.4.1 Bioinformatic processing of sequencing data

Raw sequencing data were demultiplexed based on unique barcodes and primer sequences. Paired-end reads were merged using FLASH (version 1.2.11), and residual primer and adapter sequences were trimmed with Cutadapt. Quality filtering of merged reads was conducted using fastp (version 0.23.1), resulting in high-quality clean tags. Chimeric sequences were identified and removed using the UCHIME algorithm by comparing against reference databases. Effective reads were then clustered into operational taxonomic units (OTUs) at a 97% similarity threshold using UPARSE (version 7.0.1001). The most abundant sequence in each OTU was selected as the representative sequence. Taxonomic assignment was performed using the Mothur classifier against the SILVA 138.1 database for 16S rRNA gene sequences and BLAST against the UNITE v9.0 database for ITS sequences. For unassigned amplicons, the Micro_NT reference database was used to improve annotation coverage.

### 2.5 Data analysis

Levene’s test was employed to assess variance homogeneity, while the Shapiro Wilk test was conducted to assess the normality of soil physicochemical data. Non-normally distributed data transformed: square root transformation was applied for Z-scores ranging from 2 to 3, and log10 transformation applied for Z-scores exceeding 3. One-way analysis of variance (ANOVA), followed by the Duncan’s multiple range test (α = 0.05), was utilized to identify significant differences in soil physicochemical properties across cropping systems. Variables with low overall significance were excluded to optimize subsequent analyses.

Alpha diversity indices, including Chao1, Shannon, Simpson, and ACE, were calculated using QIIME software (version 1.9.1) after rarefying all samples to an equal sequencing depth to minimize sampling bias. Statistical analysis of differences among treatments was performed using R software. Bray–Curtis distances were calculated with QIIME, and nonmetric multidimensional scaling (NMDS) visualization was generated using the ggplot2 and vegan packages in R. LEfSe analysis was employed to identify taxa with statistically significant differences in relative abundance among treatments. Taxa with an absolute LDA score > 3.0 and *p* < 0.05 were visualized in a cladogram.

Network analysis, a fundamental approach in ecological research, elucidates interactions among species within biological communities, thereby revealing ecological interdependencies and biodiversity distribution patterns. Co-occurrence networks were constructed using the “Hmisc” package, based on Spearman’s rank correlation coefficients (r) among microbial taxa. Only robust correlations (Spearman’s |r| > 0.5) with statistical significance (Benjamini–Hochberg adjusted *p* < 0.05) were retained for analysis (Fan et al., 2020; Ding et al., 2022). Network visualization was performed using Gephi version 0.9.2, where nodes represent specific OTUs and edges denote significant correlations between OTUs. Node degree, a critical topological metric, indicates the number of direct connections within the network. Network stability evaluated through iterative random node removal, assessing the robustness decline rate, which was quantified via the natural connectivity of the static network (Peng and Wu, 2016).

Mantel tests were conducted using the vegan package in R. Species composition and environmental variable matrices were transformed into distance matrices using the verdict function. Subsequently, Spearman correlations between these matrices were analyzed using the mantel function, yielding Mantel r and *p* values to assess the relationship between species composition and environmental variables.

## 3 Results

### 3.1 Changes in soil physicochemical properties

The effects of different cropping patterns on soil physicochemical properties are summarized in Table 1. Intercropping treatments (TS and TM) outperformed monoculture, although distinct differences were evident between the two intercropping systems. Compared to TT, TS significantly increased pH, CEC, TN, AP, and AK (*p* < 0.05), demonstrating its efficacy in enhancing soil nutrient availability. Compared to SS, the TS treatment elevated CEC (13.1%,) and TN (13.9%) (*p* < 0.05), respectively, while AP and AK increased markedly by 43.8 and 129.1%, respectively (*p* < 0.05). These results indicated that the tobacco–soybean intercropping facilitates nitrogen accumulation and significantly boosts the availability of fast-acting nutrients.

**Table 1:** Effects of different treatments on soil chemical properties.

Treatments	pH	CEC	SOM	TN	TP	TK	HN	AP	AK
		(cmol/kg)	(g/kg)	(g/kg)	(g/kg)	(g/kg)	(mg/kg)	(mg/kg)	(mg/kg)
TT	5.30 ± 0.01e	10.51 ± 0.23c	53.56 ± 1.00d	6.39 ± 0.04d	1.49 ± 0.02a	19.38 ± 0.13a	242.79 ± 0.56b	103.30 ± 2.31b	560.17 ± 3.74c
SS	5.67 ± 0.01b	11.87 ± 0.06b	64.35 ± 0.76a	6.93 ± 0.04b	1.34 ± 0.02c	18.62 ± 0.39b	263.88 ± 3.14a	83.12 ± 2.25c	286.50 ± 1.28d
MM	5.37 ± 0.01d	11.54 ± 0.43b	57.85 ± 1.13c	6.92 ± 0.07b	1.44 ± 0.02b	17.91 ± 0.40c	233.34 ± 1.65c	81.14 ± 2.08c	198.69 ± 1.58e
TS	5.79 ± 0.01a	13.42 ± 0.30a	61.05 ± 0.76b	7.89 ± 0.07a	1.52 ± 0.03a	19.20 ± 0.21a	267.91 ± 6.05a	119.51 ± 1.75a	656.41 ± 2.25a
TM	5.48 ± 0.01c	13.32 ± 0.30a	53.28 ± 0.79d	6.60 ± 0.03c	1.38 ± 0.03c	19.16 ± 0.20a	232.99 ± 3.86c	104.38 ± 1.61b	617.58 ± 3.74b

Different lowercase letters within the same column indicate significant differences among treatments at the *p* < 0.05 level. Values are presented as means ± standard error (*n* = 5).

Compared to TT, TM significantly increased CEC (26.7%) and AK (10.2%) (*p* < 0.05), while the increases in TN and AP were comparatively modest at 3.3 and 1.0%, respectively. Compared to MM, the TM treatment significantly enhanced CEC, TN, and AP, suggesting that tobacco–maize intercropping was more effective than MM in improving soil nutrient status. However, the effect on nitrogen accumulation was less pronounced compared to TS. When comparing the two intercropping systems, TS exhibited significantly higher TN than TM, with AP and AK levels being 14.5 and 17.2% higher, respectively (*p* < 0.05). TS better perform in soil fertility improvement, particularly in promoting nitrogen and phosphorus accumulation.

In contrast, monoculture systems demonstrated generally less soil fertility. The TT treatment exhibited the lowest CEC, TN, and AP levels. Although SS demonstrated relatively high SOM and HN levels, it displayed significantly reduced AP and AK contents (*p* < 0.05). MM observed the lowest AK content among the monoculture treatments, suggesting a relatively limited accumulation of available potassium. Overall, intercropping significantly improved soil fertility, with the TS treatment yielding the most substantial enhancement in soil nutrient status, demonstrating more pronounced benefits than TM treatment.

### 3.2 Soil microbial OTU count under different planting patterns of crops

The bacterial OTU dataset, 3,196 shared OTUs were identified across the five cropping systems, representing 32.1%. The monoculture treatments TT, SS, and MM contained 301 (3.0%), 361 (3.6%), and 488 (4.9%) unique OTUs, respectively, while the intercropping treatments TS and TM exhibited 449 (4.5%) and 685 (6.9%) unique OTUs, respectively. OTU richness was generally higher under intercropping than monoculture. TM showing the highest number of unique OTUs, indicating that intercropping, particularly TM, enhanced bacterial diversity compared to monoculture (Supplementary Figure S3a).

In the fungal OTU dataset, 656 OTUs were shared among all treatments, accounting for 12.9% of the total, a notably lower proportion than observed in the bacterial dataset. TT, SS, and MM monoculture treatments had 294 (5.8%), 517 (10.2%), and 684 (13.4%) unique OTUs, respectively. In contrast, the TS and TM intercropping treatments had 288 (5.7%) and 347 (6.8%) unique OTUs, respectively. The MM treatment demonstrated the highest number of unique fungal OTUs, while TS and TT observed the lowest (Supplementary Figure S3b).

### 3.3 Soil microbial diversity under different planting patterns of crops

Table 2 shows that different cropping patterns significantly influenced the rhizosphere soil microbial diversity. For bacterial communities, the tobacco–maize intercropping (TM) treatment exhibited the highest species richness, with significantly higher values of Observed, Chao1, and ACE indices compared to other treatments (*p* < 0.05). The average values approached 4,000, representing increases of 6.21 and 3.97% relative to tobacco monoculture (TT) and maize monoculture (MM), respectively. Although the Shannon index under TM was only slightly higher, it was significantly greater than that of TT (*p* < 0.05). Additionally, the Simpson index was significantly elevated in TM (1.98-fold higher than TT, *p* < 0.05), suggesting improved community evenness. The Pielou index was also significantly higher in TM compared to TT (*p* < 0.05).

**Table 2:** Alpha diversity indices of bacterial and fungal communities.

Microbes	Treatments	Observed	Chao1	ACE	Shannon	Simpson	Pielou
Bacteria	TT	3111.00 ± 439.75c	3745.51 ± 536.83c	3778.09 ± 543.43c	6.59 ± 0.33b	0.99 ± 0b	0.82 ± 0.03b
	SS	3528.20 ± 285.83ab	4250.09 ± 315.46ab	4264.99 ± 303.28ab	6.83 ± 0.23ab	1.00 ± 0ab	0.84 ± 0.02ab
	MM	3366.00 ± 217.34bc	4033.59 ± 252.22bc	4044.57 ± 231.00bc	6.71 ± 0.16b	1.00 ± 0ab	0.83 ± 0.01ab
	TS	3619.80 ± 270.69ab	4385.08 ± 320.58ab	4429.36 ± 332.42ab	6.88 ± 0.19ab	1.00 ± 0a	0.84 ± 0.02ab
	TM	3889.00 ± 101.30a	4668.00 ± 215.65a	4718.90 ± 211.27a	7.03 ± 0.06a	1.00 ± 0a	0.85 ± 0.01a
Fungi	TT	823.20 ± 288.02c	1081.32 ± 351.26c	1117.92 ± 367.84c	3.13 ± 0.88b	0.83 ± 0.10a	0.47 ± 0.11b
	SS	1143.40 ± 171.31ab	1438.76 ± 158.65ab	1495.49 ± 158.23ab	3.93 ± 0.78ab	0.89 ± 0.10a	0.56 ± 0.10ab
	MM	1262.60 ± 171.43a	1610.08 ± 180.53a	1658.11 ± 180.40a	4.29 ± 0.56a	0.94 ± 0.05a	0.60 ± 0.07a
	TS	876.00 ± 217.11bc	1150.71 ± 199.62bc	1210.12 ± 198.92bc	3.29 ± 0.70b	0.87 ± 0.06a	0.48 ± 0.09ab
	TM	845.40 ± 246.74bc	1108.47 ± 269.42bc	1148.19 ± 253.80c	3.12 ± 0.57b	0.87 ± 0.03a	0.46 ± 0.07b

Different lowercase letters within the same column indicate significant differences among treatments at the *p* < 0.05 level. Values are presented as means ± standard error (*n* = 5).

For fungal communities, the MM treatment had the highest Observed, Chao1, and ACE indices (*p* < 0.05), indicating the greatest fungal species richness, followed by TM, while TT and TS showed the lowest richness. Shannon and Simpson indices revealed higher diversity and evenness under SS and MM treatments, whereas the TS treatment showed the lowest Shannon index (only 3.29). Notably, the Pielou index in the TM treatment was significantly higher than in TT and TS (*p* < 0.05), indicating an improved fungal community structure under this intercropping system.

**Figure 1:**
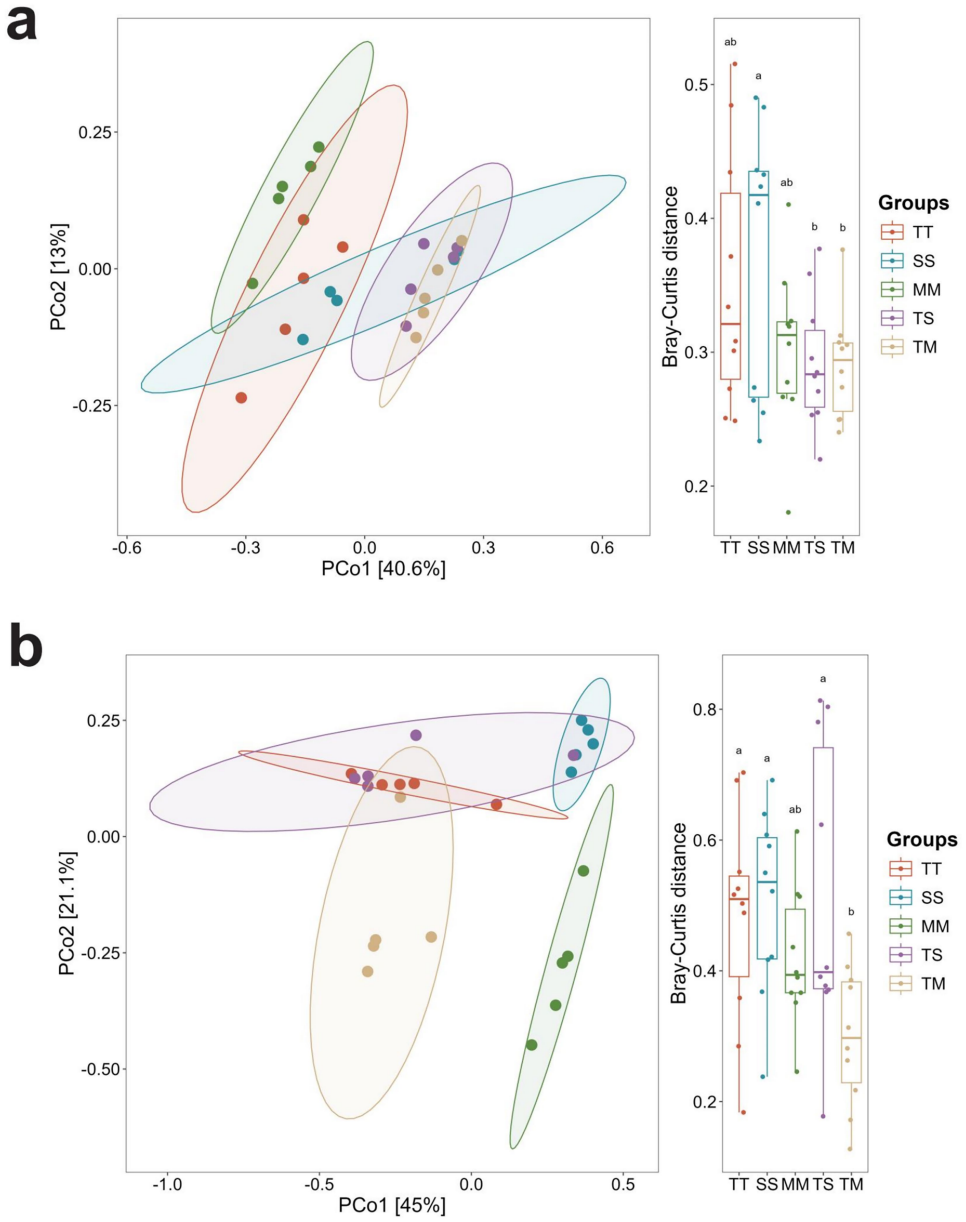
Principal coordinate analysis (PCoA) of soil microbial communities at the genus level based on Bray–Curtis distances. PCoA of soil bacterial communities at the genus level **(a)**, and PCoA of soil fungal communities at the genus level **(b)**.

Principal coordinate analysis (PCoA) based on Bray–Curtis distances showed differences in microbial community structure among treatments. For bacteria, PCo1 and PCo2 explained 40.6 and 13.0% of the total variation, respectively, with TM samples forming a relatively distinct cluster (Figure 1a). For fungi, PCo1 and PCo2 explained 45.0 and 21.1% of the variation, with TM samples also showing a clustering trend (Figure 1b). PERMANOVA analysis indicated that cropping patterns significantly affected bacterial (*R*^2^ = 0.473) and fungal (*R*^2^ = 0.5735) community composition (*p* < 0.05).

### 3.4 Soil microbial communities under different planting patterns of crops

**Figure 2:**
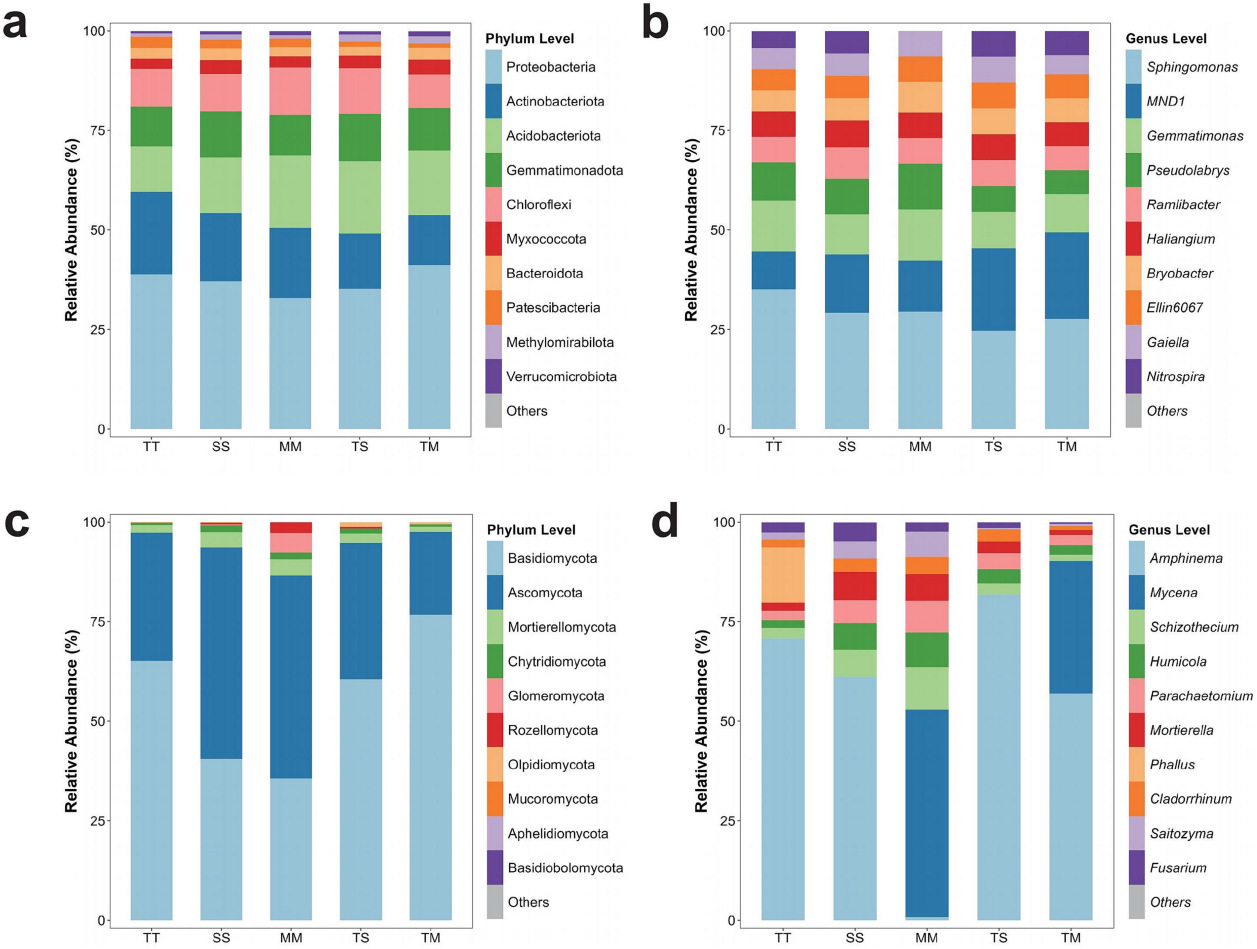
Microbial community composition at the phylum and genus levels. Bacterial community composition at the phylum level **(a)**, bacterial community composition at the genus level **(b)**, fungal community composition at the phylum level **(c)**, and fungal community composition at the genus level **(d)**.

Significant variations in the phylum-level composition of microbial communities were assessed across different cropping systems. In bacterial communities, *Proteobacteria, Actinobacteria, Acidobacteriota, Gemmatimonadota*, and *Chloroflexi* emerged as the predominant phyla in all treatments (Figure 2a). The relative abundance of *Proteobacteria* was observed as 35.2, 36.1, 33.8, 37.5, and 38.3% in TT, SS, MM, TS, and TM, respectively. *Actinobacteriota* constituted 24.7, 23.5, 26.4, 22.9, and 25.3% in the same treatments, while *Acidobacteriota* accounted for 15.1, 14.7, 13.5, 16.3, and 15.9%, respectively.

*Gemmatimonadota* displayed lower abundance in TT and SS (8.4 and 7.8%) than MM, TS, and TM (10.2, 9.9, and 10.4%, respectively) (*p* > 0.05). The relative abundance of *Chloroflexi* exhibited slight variation among treatments, ranging from 6.6 to 7.1%. An increase of about 3.4% in *Proteobacteria* abundance was observed under intercropping systems (TS and TM), but the difference was not significant (*p* > 0.05), indicating a potential facilitative effect of intercropping on this bacterial phylum. The abundance of *Actinobacteriota* was marginally higher in TM (25.3%) than TT (24.7%) but remained lower than MM (26.4%) (*p* > 0.05).

In fungal communities, *Ascomycota, Basidiomycota*, and *Mortierellomycota* were identified as the dominant phyla. The relative abundance of *Ascomycota* was 50.96, 53.00, 32.16, 34.30, and 20.78% in MM, SS, TT, TS, and TM, respectively. *Basidiomycota* accounted for 35.61, 40.56, 65.15, 60.50, and 76.75%, while *Mortierellomycota* represented 4.05, 3.86, 1.86, 2.29, and 1.23% across the same treatments (Figure 2c).

Significant variations in the genus-level composition of bacterial communities were identified across different cropping systems. *Sphingomonas* emerged as the predominant genus in all treatments, with relative abundances recorded as 35.08% (TT), 29.20% (SS), 29.47% (MM), 24.66% (TS), and 27.70% (TM) (Figure 2b). Additionally, *MND1* and *Gemmatimonas* were consistently abundant across treatments. The relative abundance of *MND1* was 9.57% (TT), 14.60% (SS), 12.82% (MM), 20.77% (TS), and 21.67% (TM), while Gemmatimonas accounted for 12.76% (TT), 10.11% (SS), 12.82% (MM), 9.09% (TS), and 9.63% (TM). A pronounced increase in *MND1* abundance was observed under TS and TM intercropping (*p* < 0.05), whereas *Sphingomonas* exhibited significantly higher abundance in TT and MM treatments (*p* < 0.05). Fungal communities exhibited substantial differences in genus-level composition depending on the cropping system (Figure 2d). *Amphinema* was consistently identified as the dominant fungal genus, with relative abundances of 70.79% (TT), 61.20% (SS), 0.79% (MM), 81.86% (TS), and 56.99% (TM). *Mycena* exhibited the highest relative abundance in MM (52.14%), followed by the TM treatment with a relative abundance of 33.25%. At the same time, it was either absent or present in minimal amounts in the other treatments (SS, TS, TT). *Mortierella* displayed higher abundance in SS (7.17%) and MM (6.72%) but exhibited a marked decline under intercropping, decreasing to 3.02% (TS) and 1.25% (TM) (*p* < 0.05). *Humicola* and *Schizothecium* were more prevalent in MM and SS (8.69 and 10.67%, respectively). However, their relative abundance diminished significantly under intercropping (TS and TM) (*p* < 0.05), indicating that monoculture systems may offer more conducive conditions for the proliferation of these fungal genera.

### 3.5 LEfSe analysis of differential microbial taxa under different cropping patterns

**Figure 3:**
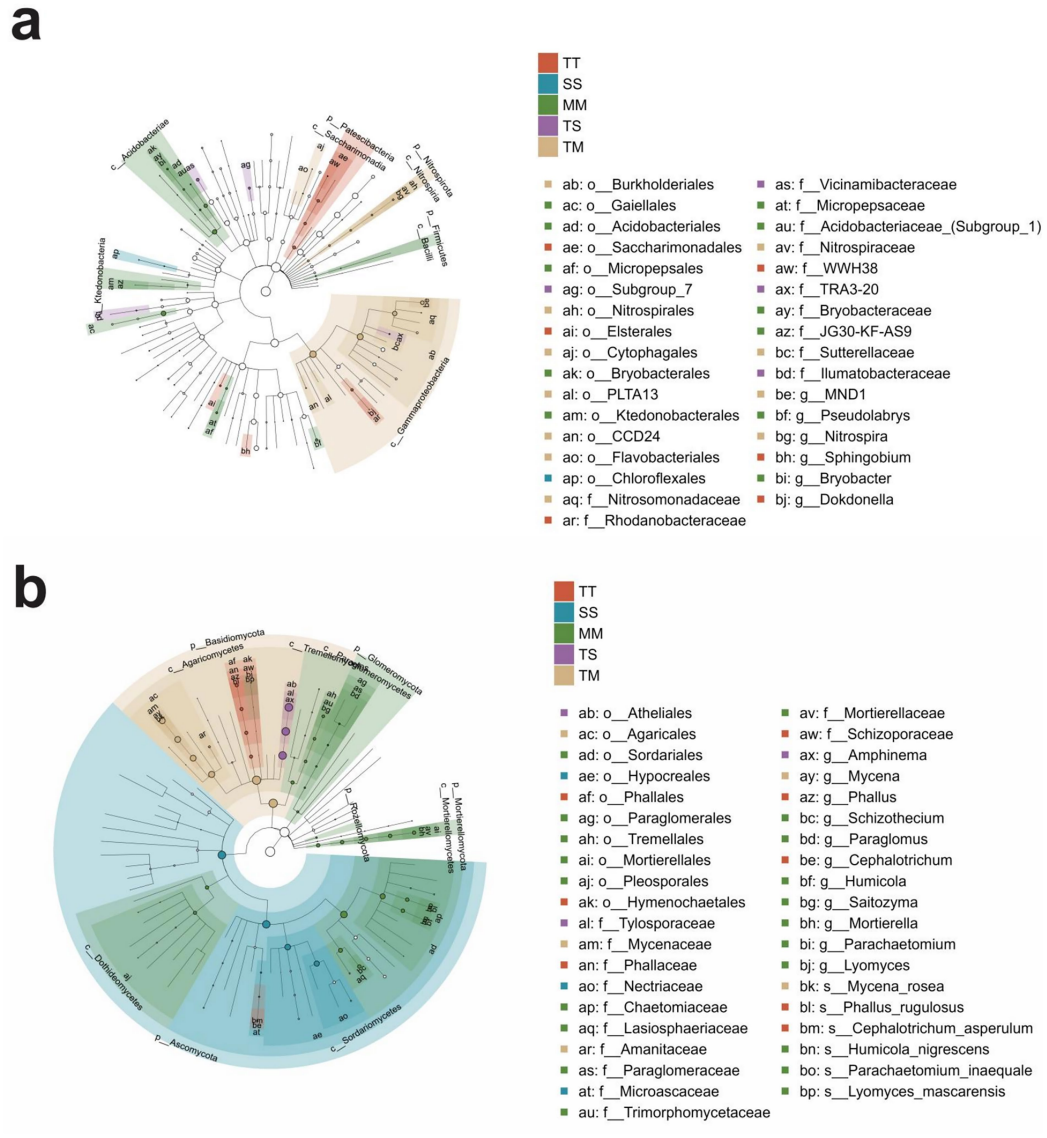
Differential taxonomic cladograms of soil bacterial and fungal communities from phylum to genus level based on LEfSe analysis. Differential taxonomic cladogram of bacterial communities from phylum to genus level **(a)**, and differential taxonomic cladogram of fungal communities from phylum to genus level **(b)**.

Based on LEfSe analysis (LDA score > 3.0 and *p* < 0.05), the top 20 core microbial taxa enriched under different cropping systems were monitored. In bacterial communities, the TM treatment was primarily characterized by the enrichment of the *Nitrosomonadaceae*, *Nitrospiraceae*, and *Sutterellaceae* families (Figure 3a). The TS treatment exhibited dominance of *Vicinamibacteraceae*, *TRA3-20*, and *Ilumatobacteraceae*, whereas *Rhodanobacteraceae*, *WWH38*, and *Ilumatobacteraceae*were predominantly enriched in the TT treatment. In contrast, the SS treatment was marked by the predominance of *Thermaceae*and, *Rhodobacteraceae*, while the MM treatment was distinguished by the enrichment of *Micropepsaceae*, *Acidobacteriaceae* (Subgroup 1), and *Bryobacteraceae*. In fungal communities, the TM treatment showed significant enrichment of *Mycenaceae*and, *Amanitaceae*. The TS treatment was dominated by the fungal families, i.e., *Tylosporaceae*, *Olpidiaceae*, and *Ceratocystidaceae*. The TT treatment exhibited a distinct predominance of *Phallaceae*, whereas the SS treatment showed higher abundances of *Nectriaceae*, *Stephanosporaceae*, and *Hydnaceae*. *Paraglomeraceae*, *Trimorphomycetaceae*, *Bryobacteraceae*, and *Glomeraceae* were most significantly enriched fungal families in the MM treatment (Figure 3b).

### 3.6 Functional characteristics of microbial communities under different planting patterns of crops

**Figure 4:**
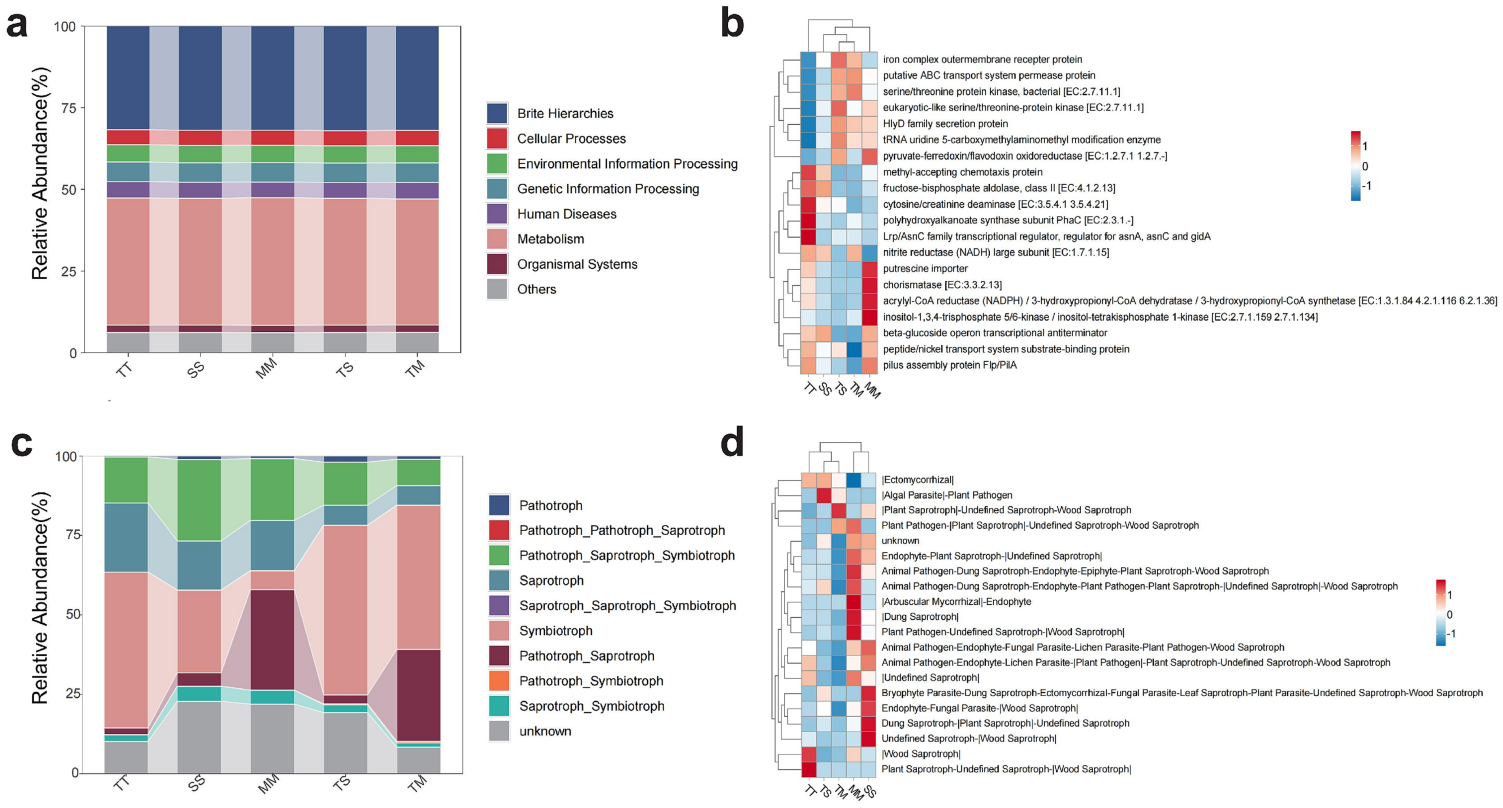
Functional prediction of soil microbial communities under different cropping systems. Predicted bacterial functions at KEGG level 1 based on PICRUSt2 **(a)**, Predicted bacterial functions at KEGG level 2, highlighting key functional gene categories **(b)**, Fungal functional guilds predicted by trophic modes using the FUNGuild database **(c)**, Relative abundance of top 20 fungal functional guilds as classified by FUNGuild **(d)**.

Based on the functional prediction results from PICRUSt2 (Figure 4a), at the first-tier functional level, the soil microbial communities were predominantly associated with metabolism-related pathways, which accounted for the largest proportion across all cropping systems. This indicates that rhizosphere microorganisms play a dominant role in fundamental metabolic processes such as nutrient transformation and carbon-nitrogen cycling. The second and third most abundant categories were Genetic Information Processing and Environmental Information Processing, suggesting that the microbial communities possess environmental responsiveness and genetic regulatory potential. In contrast, Cellular Processes and Organismal Systems were relatively less abundant, implying weaker involvement in non-essential cellular activities. Overall, there was little difference among treatments in terms of first-level functional categories; however, intercropping treatments showed slightly higher abundances in certain functions, such as genetic information processing, suggesting that diversified cropping systems may enhance microbial regulatory capacity and environmental adaptability.

Further analysis of secondary functional categories (Figure 4b) revealed that genes related to nutrient transport, such as the ABC transporter system (K02004) and iron complex outer membrane receptor protein (K02014), were most abundant across all treatments, with notably higher activity in the TM and TS intercropping systems. This highlights the central role of transmembrane transport in rhizosphere microbial functionality. Functions related to signal transduction, including eukaryotic-like serine/threonine protein kinases (K12132) and methyl-accepting chemotaxis proteins (K03406), were more enriched in the TT (tobacco monoculture) and MM (maize monoculture) treatments, reflecting enhanced microbial responses to environmental cues in monoculture conditions. Additionally, energy metabolism-related enzymes such as polyhydroxyalkanoate synthase (K03821) and fructose-bisphosphate aldolase (K01624) were more highly expressed in the TT and TM treatments, suggesting elevated carbon metabolic activity under tobacco-dominant systems. Less abundant but functionally distinctive genes such as the Lrp/AsnC family transcriptional regulator (K03718) and putrescine import protein (K14052) showed significant variation among treatments, indicating that cropping system composition influences the expression of specific microbial metabolic functions. Collectively, these results suggest that intercropping systems not only maintain core microbial functions but also enhance rhizosphere metabolic diversity and functional expression potential through plant–microbe interactions.

Functional prediction of rhizosphere fungal communities based on the FUNGuild database (Figure 4c) indicated that fungi were primarily composed of pathotrophs, saprotrophs, symbiotrophs, and their combinations. Among them, Pathotroph–Saprotroph–Symbiotroph was the dominant trophic mode, with an average relative abundance exceeding 25% in all treatments, reflecting the highly composite functional nature of rhizosphere fungal communities. Saprotrophs accounted for a large proportion, particularly enriched in the MM (maize monoculture) and TS (tobacco–soybean intercropping) treatments, suggesting a key role in organic matter degradation and nutrient turnover. Notably, symbiotrophic fungi exhibited significantly higher abundance in the TM (tobacco–maize intercropping) system, indicating that this intercropping mode may promote the enrichment of beneficial fungi such as arbuscular mycorrhizal fungi, thereby enhancing plant nutrient uptake. In contrast, pathotrophic fungi were more prevalent in the TT (tobacco monoculture) and TS treatments, potentially increasing the risk of plant diseases. Overall, tobacco–maize intercropping not only optimized fungal functional composition but may also improve the ecological services provided by soil fungi.

Significant differences in fungal functional composition were observed under different cropping patterns (Figure 4d). Analysis of the top 20 functional groups revealed that symbiotic fungi, such as ectomycorrhizal taxa, were more abundant in the TS and TM intercropping treatments, demonstrating that intercropping is conducive to the enrichment of mutualistic fungi and enhancing plant access to soil resources. Conversely, pathogen–saprotroph complexes (e.g., Plant Pathogen–|Plant Saprotroph–|Undefined Saprotroph) were more abundant in the MM and TM treatments, suggesting potential pathogenic risks alongside decomposition functions. Unclassified groups (unknown) were relatively more abundant in the SS (soybean monoculture) and MM systems, indicating that the functions of certain fungal taxa remain unclear and warrant further exploration. Additionally, multinutritional groups such as endophytes and animal pathogens were more abundant in TT and SS treatments. Collectively, the intercropping systems showed distinct advantages in enriching symbiotic fungi and optimizing fungal functional composition, highlighting the positive regulatory role of plant diversity on rhizosphere microbial functional niches.

### 3.7 Stability analysis of microbial ecological networks under different planting patterns of crops

#### 3.7.1 Microbial co-occurrence network analysis

**Figure 5:**
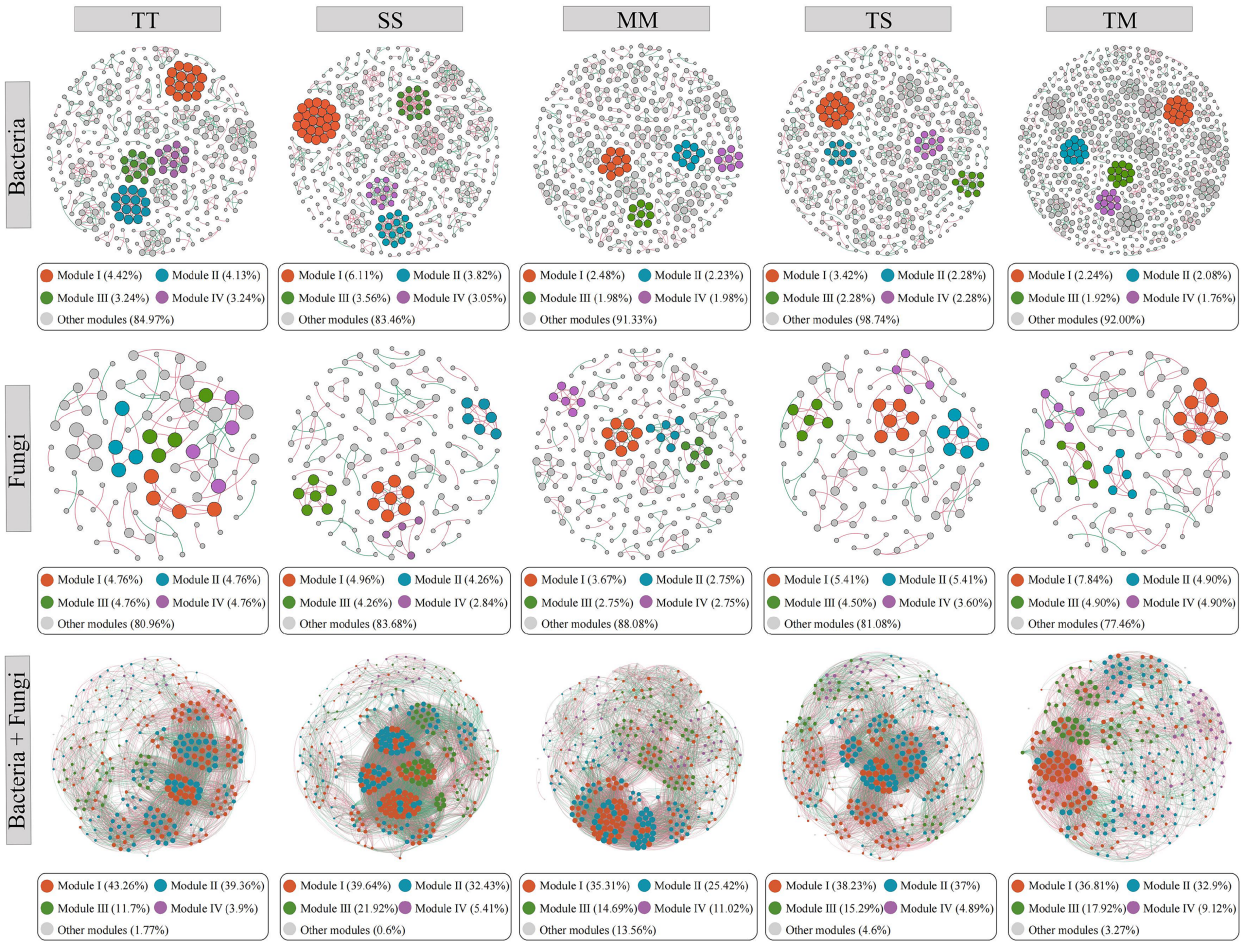
Microbial network interactions under different treatments. Nodes were colored according to different modularity classes. The size of each node is proportional to its degree. Red edges indicate positive correlations, while green edges indicate negative correlations.

Co-occurrence networks of bacterial and fungal communities were constructed under TT, SS, MM, TS, and TM cropping treatments to elucidate the impact of cropping patterns on microbial network structure (Figure 5; Supplementary Table S1). The bacterial network analysis revealed that the TM treatment exhibited the highest number of nodes (625) and edges (1,516), indicating that tobacco–maize intercropping substantially enhanced the complexity and interaction intensity within the bacterial network. The high modularity score (0.979) further suggested the improved functional robustness within this network. The TS treatment exhibited a higher number of nodes (439), edges (991), and modularity (0.969) compared to the three monoculture treatments, and its average degree (4.51) was close to that of the TM treatment, indicating that tobacco–soybean intercropping also contributed to strengthening bacterial interactions and maintaining a well-structured network. In contrast, the MM treatment displayed significantly lower number of nodes (404) and edges (688), along with reduced average degree (3.41), indicating that maize monoculture led to sparser bacterial community and diminished network connectivity. The SS treatment demonstrated the highest average degree (6.39), indicating that soybean monoculture intensified network density by reinforcing node interactions. The TT treatment exhibited moderate network density (0.014) and heterogeneity (0.780), implying that tobacco monoculture may contribute to sustaining the stability of core nodes within the bacterial community.

Fungal network analysis also revealed pronounced effects of cropping patterns. The TM treatment presented the highest number of nodes (102), edges (137), and network density (0.027), indicating that tobacco–maize intercropping significantly enhanced the complexity and tightness of the fungal network. The TM treatment exhibited the highest centralization (0.043), indicating an increased influence of key nodes within the network. The TS treatment exhibited higher numbers of nodes (111), edges (118), and network density (0.019) compared to the TT treatment, indicating that tobacco–soybean intercropping also contributed to enhancing the connectivity and compactness of the fungal network. Its centralization (0.026) and heterogeneity (0.619) were also relatively higher, suggesting a better balance between maintaining core community structure and supporting fungal diversity under this treatment. The SS treatment displayed the most significant number of nodes (141) and heterogeneity (0.713), suggesting that soybean monoculture fostered greater fungal community diversity, though the lower network density (0.015) indicated more fragmented connectivity. The fungal network of the TT treatment showed the lowest number of nodes (84) and edges (78), characterized by low density (0.022) and heterogeneity (0.451), implying that tobacco monoculture may limit fungal network complexity. Modularity analysis consistently demonstrated high modularity values (> 0.92) across bacterial and fungal networks for all treatments. Bacterial networks, particularly under MM and TM treatments (both 0.979), displayed exceptionally high modularity, indicating that modularity may be crucial for maintaining network stability. Although the modularity of fungal networks was comparatively lower, it still demonstrated strong ecological compartmentalization.

The integrated co-occurrence network constructed from both bacterial and fungal communities further demonstrated that cropping patterns had a substantial impact on microbial interaction structures (Figure 5; Supplementary Table S2). The SS treatment exhibited the largest network size, with 333 nodes and 10,395 edges. It also had the highest average degree (62.43) and network density (0.19), indicating that soybean monoculture promoted extensive co-occurrence between bacteria and fungi, thereby significantly enhancing microbial network connectivity and complexity. In contrast, the TM treatment had the lowest number of nodes (307), edges (5,634), and average degree (36.70), along with the lowest network density (0.12) and modularity (1.43). This suggests that although tobacco–maize intercropping enhanced interaction intensity within bacterial or fungal networks individually, the overall structure of the integrated network was relatively loose, possibly due to niche differentiation or reduced complementarity between microbial taxa. The TT, TS, and MM treatments showed intermediate network properties. TT exhibited a network density of 0.17 and modularity of 6.56, suggesting that tobacco monoculture helped maintain a relatively compact community structure with strong functional modularity. Although MM had the highest number of nodes (345), its average degree (44.84) and modularity (3.51) were relatively low, indicating weaker connectivity and modular organization within the microbial co-occurrence network under maize monoculture. The TS treatment showed a network structure similar to TT, characterized by a favorable clustering coefficient (0.62) and average path length, indicating that tobacco–soybean intercropping contributed to maintaining stable microbial interactions.

#### 3.7.2 Natural connectivity analysis

**Figure 6:**
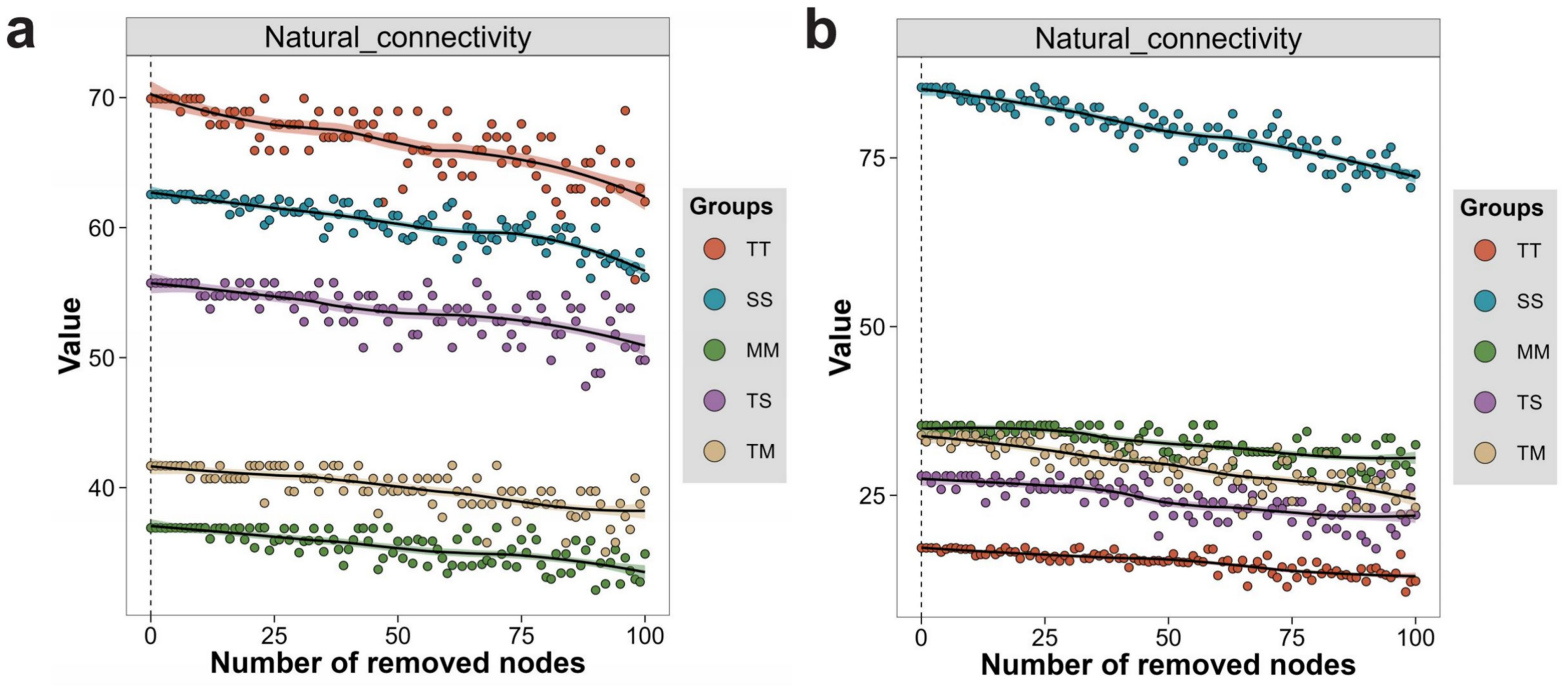
Changes in microbial network natural connectivity with increasing node removal under different treatments. Changes in natural connectivity of bacterial networks under different treatments during sequential node removal **(a)**, and changes in natural connectivity of fungal networks under different treatments during sequential node removal **(b)**.

Natural connectivity analysis revealed significant differences in microbial co-occurrence networks’ stability and disturbance resistance. In the bacterial network, the TT treatment exhibited the highest natural connectivity and experienced the smallest decline during node removal, indicating that tobacco monoculture enhanced network stability. The SS treatment demonstrated the second-highest natural connectivity, suggesting that soybean monoculture improved network robustness by strengthening interactions among key nodes (Figure 6a). In contrast, the MM and TM treatments displayed the lowest natural connectivity with the steepest decline, indicating that bacterial networks under maize monoculture and tobacco–maize intercropping were more vulnerable to disturbance, likely due to their sparse structure and decentralized key nodes. The TS treatment presented intermediate values, reflecting moderate increase in resistance to disturbance resulting from tobacco–soybean intercropping. In the fungal network, the SS treatment exhibited significantly higher natural connectivity and smaller decline during node removal, indicating the strongest network stability. This finding suggests that soybean monoculture enhances fungal network robustness by providing stable resource support. Conversely, the TT treatment demonstrated the lowest natural connectivity, indicating that tobacco monoculture constrained the fungal network’s capacity to withstand perturbation. Although TS and TM treatments showed lower initial connectivity, they exhibited increased buffering capacity during the later stages of node removal. Notably, the TM treatment displayed a gradual decline rate, indicating that intercropping improved network stability by enhancing modularity and structural complexity (Figure 6b).

Cropping treatments significantly impacted the structure and natural connectivity of bacterial and fungal networks. Monoculture treatments (TT and SS) exhibited higher initial connectivity and network stability within their microbial communities. In contrast, intercropping treatments (TS and TM) demonstrated enhanced buffering capacity under disturbance by promoting network complexity and modular organization.

### 3.8 Relationships between soil microbial communities and soil physicochemical properties under different planting patterns of crops

**Figure 7:**
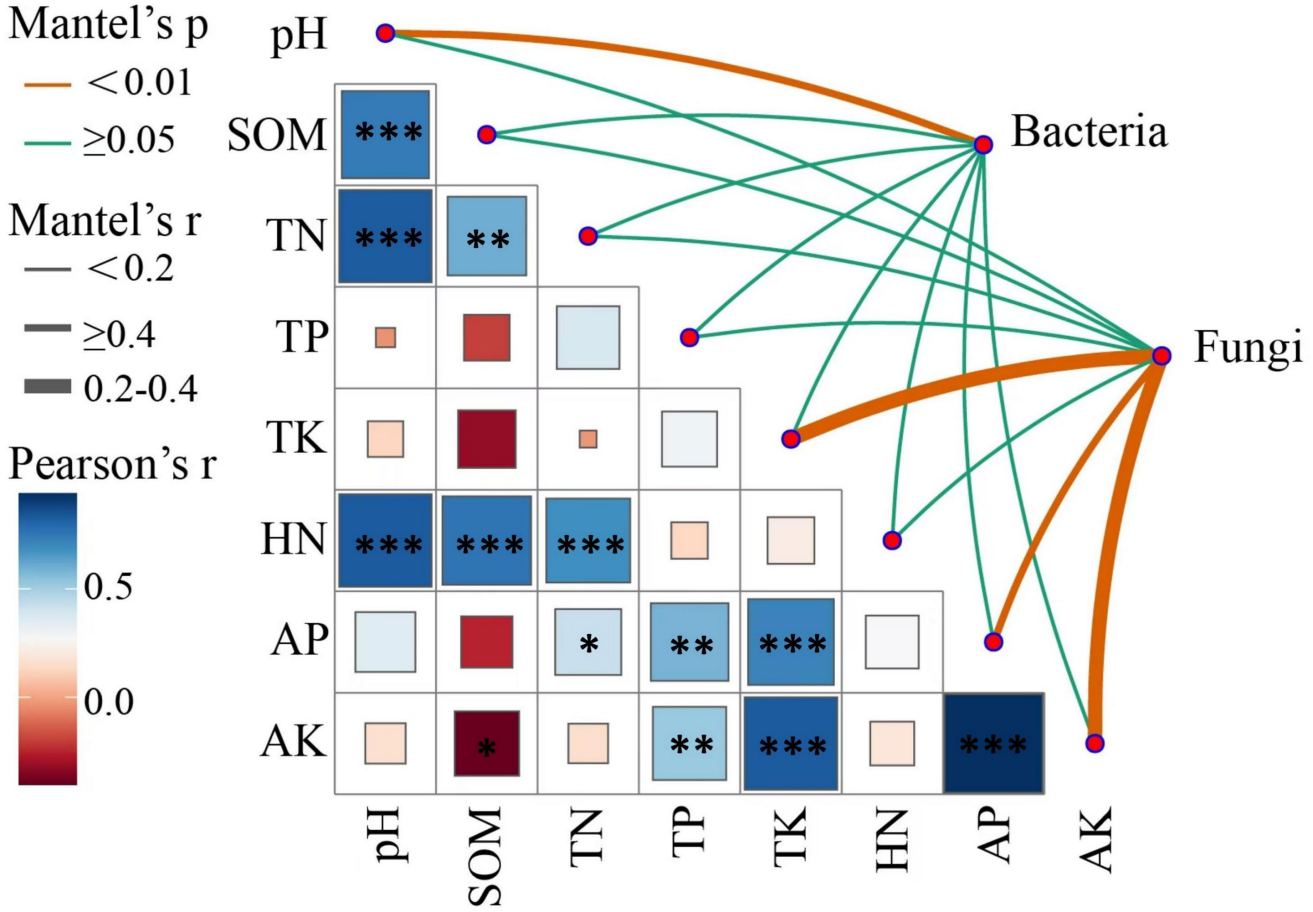
Mantel analysis of the relationships between soil environmental factors and microbial community structure under different cropping systems.

Mantel tests were conducted to assess the correlations between soil chemical properties and the composition of bacterial and fungal communities (Figure 7; Supplementary Table S3). In bacterial communities, soil pH positively correlated with community composition (Mantel, *r* = 0.21, *p* = 0.006), indicating highly significant relationship. In contrast, other soil chemical properties, including SOM, TN, TP, TK, HN, AP, and AK, showed low Mantel *r* values and did not correlate significantly with bacterial community composition (*p* ≥ 0.05). In fungal communities, TK (Mantel, *r* = 0.485, *p* = 0.001), AP (Mantel, *r* = 0.373, *p* = 0.001), and AK (Mantel, *r* = 0.494, *p* = 0.001) demonstrated highly significant positive correlations with community composition. Conversely, pH, HN, SOM, TN, and TP did not correlate significantly with fungal community composition (*p* ≥ 0.05). These results indicated that the soil pH is critical environmental factor influencing bacterial community composition, while TK, AP, and AK significantly shape fungal community structure. These insights contribute to deeper understanding of how soil chemical properties regulate microbial community dynamics.

## 4 Discussion

### 4.1 Soil physicochemical properties

Compared to monoculture systems, tobacco–soybean and tobacco–maize intercropping significantly enhanced the soil CEC, TN, AP, and AK level, likely due to improved nutrient cycling and increased nutrient use efficiency (NUE), thereby promoting soil fertility (Pariz et al., 2020; Shu et al., 2024). Additionally, intercropping significantly increased soil pH compared to tobacco monoculture, positively influencing soil acid–base balance (Cong et al., 2015; Wu et al., 2016). However, the extent of nutrient improvement varied between intercropping systems. Tobacco–soybean intercropping proved more effective in achieving balanced accumulation of N, P, and K, primarily attributable to the nitrogen-fixing capacity of soybean. As a leguminous crop, soybean forms symbiotic associations with rhizobia, which fixes atmospheric nitrogen, thus providing additional nitrogen source to the soil (Yong et al., 2018; Liu et al., 2021). This process significantly increases total and available nitrogen levels.

Furthermore, organic acids secreted by soybean roots can mobilize insoluble phosphate, enhancing available phosphorus content and improving soil phosphorus availability (Hinsinger, 2001). In contrast, tobacco–maize intercropping was more effective in increasing available potassium content. Maize, known for its high potassium uptake efficiency, activates soil potassium during growth, converting more mineral potassium into plant-available forms, thus boosting the soil potassium-supplying capacity (Zörb et al., 2014). Moreover, previous studies have shown that the maize rhizosphere is enriched with potassium-solubilizing bacteria, which can effectively promote the activation and transformation of mineral potassium in the soil, thereby increasing the content of available potassium and its accessibility to plants (Etesami et al., 2017). These microorganisms solubilize insoluble potassium from clay minerals through mechanisms such as the secretion of organic acids, chelators, and the formation of biofilms, converting it into more exchangeable or water-soluble forms (Ahmad et al., 2016). Compared to monoculture systems, intercropping significantly improved soil physicochemical properties, enhancing soil fertility. Tobacco–soybean intercropping exhibited better performance accumulating nitrogen, phosphorus, and potassium, indicating its potential suitability for improving nutrient conditions in tobacco fields.

### 4.2 Microbial community structure and diversity analysis

Long-term monoculture often reduces soil microbial diversity, whereas intercropping systems can increase microbial species richness and diversity. This study demonstrated that the intercropped soils exhibited significantly higher bacterial diversity and evenness than monoculture systems, with tobacco–maize intercropping having the most pronounced positive effect on bacterial species richness. These results align with previous findings indicating that intercropping enhances bacterial diversity (Li and Wu, 2018; Wang et al., 2023). However, contrary to previous reports suggesting that intercropping enhances fungal diversity (Peng et al., 2014; Mwakilili et al., 2021), fungal diversity was notably higher in monoculture maize and soybean systems compared to tobacco–maize and tobacco–soybean intercropping systems. Among the intercropping treatments, tobacco–soybean exhibited the lowest fungal diversity, indicating potential suppression of specific fungal communities. However, tobacco–maize intercropping improved fungal community structure, and the enhancement in diversity remained lower than monoculture treatments. This phenomenon may be attributed to the influence of tobacco root exudates. Previous studies have reported that the amino acids secreted by tobacco roots can inhibit the growth of specific fungal taxa (Li et al., 2024), which may explain the reduced fungal diversity in intercropping systems involving tobacco.

PCoA analysis revealed distinct differences in bacterial community composition between intercropping and monoculture systems, indicating that intercropping significantly altered the soil bacterial community structure. These changes can be attributed to intercropping-induced modifications in soil nutrient availability and moisture conditions, which subsequently influence bacterial community dynamics (Gao et al., 2019). *Proteobacteria*, *Actinobacteria*, and *Acidobacteriota* emerged as the dominant phyla across all treatments, playing critical roles in soil nutrient cycling and carbon and nitrogen metabolism (Li et al., 2022). Notably, the relative abundance of *Proteobacteria* was higher under intercropping than monoculture systems, indicating that intercropping may facilitate the proliferation of this phylum. This observation aligns with the known functional traits of *Proteobacteria*, including efficient nutrient uptake and pathogen defense, which are advantageous under intercropping conditions. *Actinobacteriota* abundance was remarkably increased in the TM treatment, likely due to the antibiotic-producing and organic matter decomposing capabilities of *Actinobacteria*. Intercropping may enhance organic matter content and modify the soil microbial structure, creating favorable ecological niches that support actinobacterial proliferation. At the genus level, *Sphingomonas* remained dominant across all treatments, with a notably higher relative abundance under monoculture systems, whereas its prevalence decreased under intercropping. As a genus known for its adaptability and involvement in carbon and nitrogen metabolism, *Sphingomonas* typically thrives in stable environments, such as monoculture systems.

In contrast, the increased soil heterogeneity under intercropping likely introduces competitive interactions with other bacterial taxa, diminishing the relative abundance of *Sphingomonas*. Conversely, the genus *MND1* exhibited significant increase in relative abundance under intercropping, suggesting that intercropping supports the enrichment of this nitrogen-cycling group. *MND1* is predominantly involved in ammonia oxidation and nitrification, with its functional role varying between intercropping treatments in the TS treatment. It primarily participates in organic nitrogen transformation, while in the TM treatment, it is more closely associated to nitrification processes.

In fungal communities, intercropping significantly elevated the relative abundance of *Basidiomycota*, a phylum integral to soil organic matter decomposition and carbon cycling. This increase indicates that intercropping positively influences soil carbon transformation efficiency. *Mycena* was detected exclusively in the MM and TM treatments at the genus level, which may be associated with its known negative correlation with soil pH. The lower pH observed in these treatments likely contributed to the increased relative abundance of *Mycena* (Feng et al., 2024). The saprotrophic genus *Humicola* showed higher relative abundance under monoculture conditions but declined significantly under intercropping. This reduction suggests that the intercropping optimized the soil environment, reducing the dependence on saprotrophic fungi for organic matter decomposition and fostering the development of microbial communities more conducive to efficient nutrient cycling (Lian et al., 2019).

### 4.3 Functional characteristics of microbial communities

LEfSe analysis revealed that the intercropping systems (TS and TM) significantly recruited more core microbial taxa related to nutrient cycling and symbiotic interactions by optimizing the soil environment, thereby potentially improving nutrient use efficiency and enhancing plant health and stress resilience (You et al., 2024; Su X. et al., 2024; Su D. et al., 2024). For example, the enrichment of ammonia-oxidizing bacteria (*Nitrosomonadaceae*) and nitrifying bacteria (*Nitrospiraceae*) in the TM treatment suggests that this system may enhance nitrogen mineralization and nitrification processes, thus improving nitrogen utilization efficiency in soil (Liu et al., 2022). In the TS treatment, the recruitment of functional microbes such as symbiotic fungi (*Tylosporaceae*) and organic matter-degrading bacteria (*Ilumatobacteraceae*) may strengthen rhizosphere microbial interactions and promote synergistic functioning and ecological stability of the micro-ecosystem (Lian et al., 2019). In contrast, monoculture systems (TT, SS, MM) tended to enrich certain potential pathogenic or anaerobic microbial groups. For instance, *Nectriaceae* (including Fusarium) enriched in the SS treatment are typical plant pathogens that have been reported to be associated with root rot diseases in various crops (Todorović et al., 2023). *Rhodanobacteraceae* and *Bryobacteraceae*, dominant in TT and MM treatments, are commonly found in oxygen-deficient and reductive soil environments, which may reflect soil degradation and increased disease risk under monoculture practices (Green et al., 2012). Notably, the increased abundance of arbuscular mycorrhizal fungi (*Glomeraceae*) in MM treatment may represent a plant adaptation strategy to nutrient deficiency, facilitating the acquisition of poorly soluble nutrients such as phosphorus and potassium through symbiotic mechanisms (Akpodé et al., 2023). In summary, the intercropping system not only promotes the enrichment of beneficial microbes by modulating microbial community structure, but also suppresses potential pathogens to a certain extent, thereby optimizing soil ecological functions and enhancing the stability and resilience of the crop system (Zhao et al., 2022).

### 4.4 Stability analysis of microbial ecological networks

Microbial community stability is generally positively correlated with structural complexity (Girvan et al., 2005; Wagg et al., 2014). Higher species diversity is often accompanied by optimized functional redundancy and ecological niche differentiation, and the high stability of microbial communities is a crucial foundation for sustaining ecosystem functions (Girvan et al., 2005). In the present study, intercropping significantly enhanced both the complexity and interaction strength of bacterial and fungal co-occurrence networks. This may be attributed to the diversified cropping systems increasing resource heterogeneity and niche diversity in the rhizosphere microenvironment. Differences in the type and quantity of root exudates among different crops may promote the coexistence of functionally complementary or antagonistic microbes, facilitating the establishment of complex relationships such as symbiosis and competition (Barberán et al., 2012; Gao et al., 2022). Moreover, previous studies have shown that intercropping can alleviate continuous cropping obstacles and enhance spatial heterogeneity and carbon–nitrogen inputs, thereby strengthening ecological connections and functional redundancy among microorganisms. These effects jointly lead to the development of more modular and disturbance-resilient network structures (Philippot et al., 2013). Therefore, intercropping systems may reshape soil environments and rhizosphere ecological processes, driving ecological reorganization of microbial community structures and thereby enhancing network complexity and robustness. Beyond complexity, modularity is regarded as a key topological feature in maintaining microbial community stability and ecological functional diversity. Modularity reflects the degree of functional compartmentalization within a network, i.e., the extent to which the network can be divided into tightly interconnected subgroups with relatively sparse connections between them. Such structures can localize the impact of disturbances, thus increasing the overall robustness of the network (Stouffer and Bascompte, 2011; Coyte et al., 2015). In this study, the bacterial networks in the tobacco–maize (TM) and tobacco–soybean (TS) intercropping treatments exhibited significantly higher modularity indices (0.979 and 0.969, respectively), indicating clearer functional partitioning and coordinated interactions, which likely reflect microbial responses to rhizosphere spatial heterogeneity. Combined with the natural connectivity analysis, the intercropping treatments showed a slower decline in connectivity upon sequential node removal, further confirming the structural elasticity and functional redundancy advantages of highly modular networks in resisting external perturbations. In contrast, although monoculture treatments showed slightly higher initial connectivity, their networks were more dependent on keystone taxa. Once these key nodes were lost, the networks were more prone to fragmentation, revealing their limited resilience to disturbances (Li et al., 2025). From the perspective of network topology, intercropping systems enhance microbial community modularity and functional compartmentalization, thereby increasing the buffering capacity against environmental stress. This represents an effective strategy for constructing resilient agricultural microbial networks.

### 4.5 Relationships between microorganisms and soil physicochemical properties

Soil physicochemical properties are key drivers of changes in microbial community structure (Sui et al., 2021; Wang A. et al., 2024; Wang G. et al., 2024). Among them, pH, organic matter, and the availability of nitrogen and phosphorus have been widely recognized as major environmental factors shaping microbial community composition (Geisseler and Scow, 2014; Delgado and Gómez, 2024). In this study, a significant correlation was observed between soil pH and bacterial community composition. Previous research has shown that pH directly affects microbial physiological processes in soil and modulates the dominance of specific bacterial taxa, while the influence of nutrient factors may be indirectly regulated by pH shifts (Romanowicz et al., 2016). Rousk et al. (2010) further demonstrated that pH can constrain the survival and adaptability of different bacterial groups by altering membrane stability, extracellular enzyme activity, and nutrient solubility. In contrast, organic matter, total nitrogen, and total phosphorus showed no significant correlations with bacterial community composition in this study, likely due to the overriding effect of pH. Several soil gradient studies have also confirmed that when large pH differences exist, the influence of other physicochemical factors is often masked by pH (Fierer and Jackson, 2006; Lauber et al., 2009). This pattern was also observed in our results, underscoring the critical ecological role of pH in shaping bacterial community structure.

In contrast, we found that the structure of fungal communities was more strongly associated with total potassium, available potassium, and available phosphorus, suggesting a higher sensitivity of fungi to mineral nutrient availability. This is in agreement with findings from various ecosystems. For instance, Wu et al. (2022) demonstrated through Mantel tests that available phosphorus was the dominant factor shaping fungal community composition in wheat-cultivated soils under long-term fertilization. Similarly, Zhang et al. (2022) reported that available potassium was the primary variable influencing fungal community dynamics in a biochar-amended yellow limestone soil. Studies in forested wetland ecosystems also emphasized the role of available potassium in regulating fungal spatial distribution (Geng et al., 2023; Lekberg et al., 2021). These patterns may arise from the distinct ecological functions of fungi in nutrient cycling. Arbuscular mycorrhizal fungi enhance plant uptake of phosphorus and potassium through symbiotic associations, while saprotrophic fungi decompose organic matter and promote the mineralization and release of potassium. The niche differentiation between these two functional groups becomes evident along nutrient gradients. In nutrient-rich soils, saprotrophic or pathogenic fungi adapted to high nutrient conditions tend to dominate and rapidly restructure the community (Wang et al., 2023, 2025); conversely, in nutrient-poor environments, symbiotic fungi that rely on mycorrhizal associations may prevail (Yang K. et al., 2025; Yang H. et al., 2025). Notably, pH did not significantly affect fungal communities in this study, which aligns with their broader tolerance range: pure culture experiments indicate that most fungi can grow normally across a pH range of 4–9 (Rousk et al., 2010), making them less sensitive to pH fluctuations compared to bacteria. Therefore, in soils with limited pH variation but distinct nutrient gradients, mineral nutrients such as phosphorus and potassium are likely to be the dominant drivers of fungal community assembly (Yan et al., 2024).

### 4.6 Ecological applicability and future research perspectives

In summary, this study, which focused on tobacco intercropping systems, reveals an ecological regulation mechanism wherein crop diversification enhances the stability of microbial networks by optimizing soil physicochemical conditions. These findings demonstrate promising potential for application beyond major flue-cured tobacco production regions. This mechanism shows broad applicability, particularly in agricultural ecosystems facing challenges such as continuous cropping obstacles and soil degradation. Using microbial network complexity and functional stability as key indicators may offer a microecological basis for optimizing cropping structures and developing sustainable agricultural models. While further application in other systems such as medicinal plants, fruits and vegetables, and economic forests should be adapted to local conditions, this study provides theoretical support and practical reference for the microecological regulation of farmland, with potential for cross-regional adoption. It should be noted that this study was based on short-term, single-season observations and did not capture long-term dynamics of microbial communities under different climatic conditions; thus, the temporal stability of the observed effects requires further validation. Moreover, although the study focused on soil-level ecological responses, field observations suggested that tobacco–maize intercropping may pose potential quality concerns during harvest due to maize pollen adhesion or leaf shading effects. This issue was not fully addressed in the current study. Future research should incorporate physiological and biochemical assessments of tobacco leaves to better understand how interspecific interactions influence commercial and sensory quality, thereby improving the comprehensive evaluation framework for intercropping systems.

## 5 Conclusion

This study systematically revealed that tobacco intercropping plays a critical role in enhancing soil nutrient supply capacity and ecosystem stability by regulating microbial community structure and increasing the complexity of co-occurrence networks. Specifically, tobacco–soybean intercropping significantly improved the availability of soil nitrogen and phosphorus through biological nitrogen fixation and phosphorus mobilization, while tobacco–maize intercropping enriched key functional taxa such as Actinobacteria, thereby enhancing the modularity and robustness of microbial networks and improving the soil system’s resilience to external disturbances. The findings demonstrate that crop diversification can optimize the soil physicochemical environment and restructure microbial communities, leading to the formation of stable and efficient microbial ecological networks. This strategy has broad applicability, particularly in agricultural ecosystems facing challenges such as continuous cropping obstacles and soil degradation. Using microbial community structure and functional stability as evaluation indicators may provide a theoretical basis for optimizing cropping systems and developing sustainable agricultural models. Overall, this study offers microecological insights and practical guidance for mitigating the risks of continuous tobacco cropping, enhancing soil ecological resilience, and promoting sustainable agricultural development.

## Data Availability

The datasets presented in this study can be found in online repositories. The names of the repository/repositories and accession number(s) can be found in the article/Supplementary material.
