# Supplementary material for: Tobacco intercropping enhances soil fertility by improving synergic interactions between soil physicochemical and microbial properties
Source: Front Microbiol. 2025 Sep 2;16:1647493. doi: 10.3389/fmicb.2025.1647493 (PMC12436390; doi:10.3389/fmicb.2025.1647493)
Supplement: Supplementary file 1 [file Data_Sheet_1.docx]

**Types of paper:** Original Research

**Tobacco Intercropping enhances soil fertility by improving Synergic Interactions between Soil Physicochemical and Microbial Properties**

Kaiyuan Gu^1,2,†^, Xianglu Liu^1,†^, MingLiu^1,2^, Xu Wei^1^, Juan Li^2^, Yanxia Hu^2^,Yonglei Jiang^3^, Yi Chen^3^, Dexun Wang^2^, Yanming Yang^2^, Jiaen Su^2,⁎^, Longchang Wang^1,⁎^

^1^College of Agronomy and Biotechnology, Southwest University/ Engineering Research Center of South Upland Agriculture, Ministry of Education, Chongqing, 400715, China

^2^Yunnan Tobacco Company, Dali State Branch, Dali, 671000, Yunnan, China

^3^Yunnan Academy of Tobacco Agricultural Sciences, Kunming, 650021, Yunnan, China

^†^These two authors contributed equally to this article.

^⁎^Corresponding authors

Emails: [dlyc8816@163.com (Jiaen Su)](mailto:dlyc8816@163.com%20(Jiaen%20Su)), [wanglc2003@163.com (Longchang Wang)](mailto:wanglc2003@163.com%20(Longchang%20Wang))

**Supplementary**

**Notes S1** Site description and experimental design

**Figure S1** Schematic diagram of intercropping patterns

**Figure S2** Field layout and design diagram

**Figure S3** Venn Diagram of Soil Bacterial and Fungal OTUs in Tobacco Intercropping Systems

**Figure S4** Box Plot of Rhizosphere Microbial Diversity in Tobacco Intercropping Systems

**Table S1** Topological Properties of Microbial Networks Across Different Treatments

**Table S2** Mantel Analysis of the Relationships Between Soil Environmental Factors and Microbial Community Structure Under Different Cropping Systems

**Note S1**. Site description and experimental design

This experiment was conducted in 2023 and 2024 in Midu County, Dali Bai Autonomous Prefecture, Yunnan Province (25.38˚N, 100.41˚E), using a randomized block design. Five treatments were set: TT (tobacco monoculture), SS (soybean monoculture), MM (maize monoculture), TS (tobacco and soybean intercropping with a row ratio of 1:4), and TM (tobacco and maize intercropping with a row ratio of 1:1). Each treatment included 5 replicate plots, with an area of 23.29 square meters per plot. The tobacco variety used was Honghua Dajin Yuan, the soybean variety was Yunhuang 13, and the maize variety was Jiyuan 8. The planting arrangements in the monoculture and intercropping plots are as follows: In the monoculture plots, tobacco was planted at a spacing of 50 cm × 120 cm, yielding a planting density of 16,500 plants per hectare; maize monoculture had a planting density of 60,000 plants per hectare, while soybean monoculture reached a density of 330,000 plants per hectare. The specific layout of the intercropping patterns is depicted in Figure S1. In the intercropping treatments, the planting density of tobacco was maintained at the same level as in the monoculture plots. For the TS treatment (tobacco–soybean intercropping), tobacco and soybean were intercropped at a row ratio of 1:4, with soybean seeds sown on both sides of the tobacco row, 20 cm from the base of the tobacco plants. The soybean rows were spaced 10 cm apart, with an intra-row spacing of 25 cm between soybean plants, resulting in a planting density of approximately 118,800 plants per hectare. In the TM treatment (tobacco–maize intercropping), tobacco and maize were intercropped at a row ratio of 1:1, with maize seeds sown on one side of the tobacco row, 40 cm from the base of the tobacco plants. The planting density of maize was identical to that of tobacco, both set at 16,500 plants per hectare.In terms of experimental arrangements, tobacco seedlings were floated from February 22 to 23 each year, transplanted from May 1 to 3, topped from July 17 to 21, and harvested from September 10 to 12. Both soybean and maize were sown from May 16 to 20 each year, with soybean harvested from August 23 to 26 and maize harvested from September 8 to 14. In terms of fertilization management, tobacco was uniformly fertilized with tobacco-specific fertilizer (N: P: K = 10: 10: 24) before transplanting, at a rate of N: 60 kg·ha⁻¹, P₂O₅: 60 kg·ha⁻¹, and K₂O: 144 kg·ha⁻¹. After transplanting, tobacco was top-dressed every 30 days, with two applications, each applying N: 15 kg·ha⁻¹, P₂O₅: 15 kg·ha⁻¹, and K₂O: 36 kg·ha⁻¹. Maize was fertilized with maize-specific compound fertilizer (N: P: K = 30: 9: 6) at 600 kg·ha⁻¹ as the base fertilizer, and urea (45%) was applied at 120 kg·ha⁻¹ during the tassel stage. Soybean was fertilized with soybean-specific water-soluble compound fertilizer (N: P: K = 13: 17: 15) at 165 kg·ha⁻¹ as the base fertilizer, and water-soluble compound fertilizer (N: P: K = 10: 10: 10) was applied at 165 kg·ha⁻¹ during the pod-setting stage. After harvest, all plots were winter-tilled to prevent the impact of crop rotation. All crops in this experiment were irrigated by rainwater. All management practices had been maintained at the beginning of the experiment.


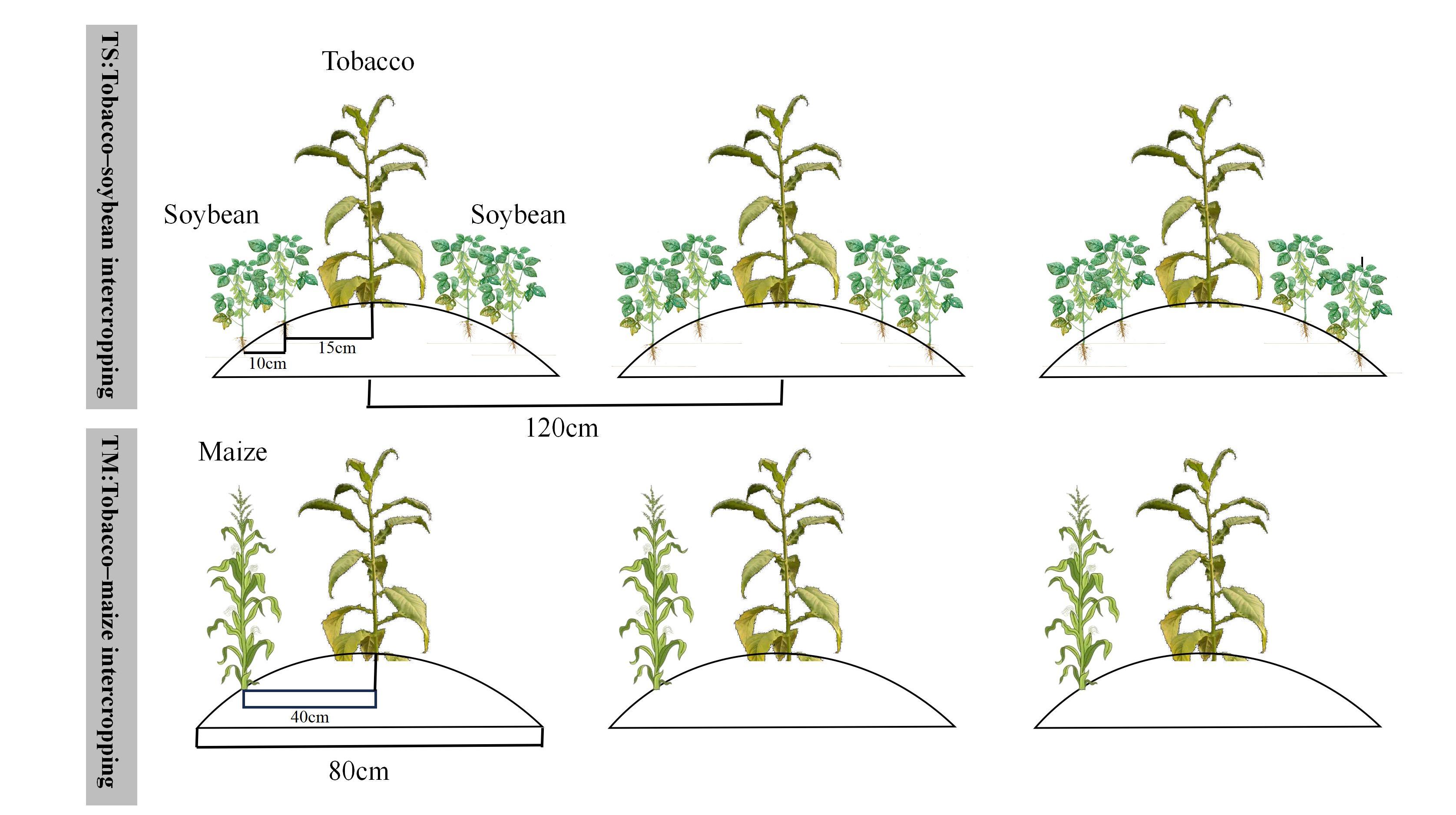


Figure S1. Schematic diagram of intercropping patterns


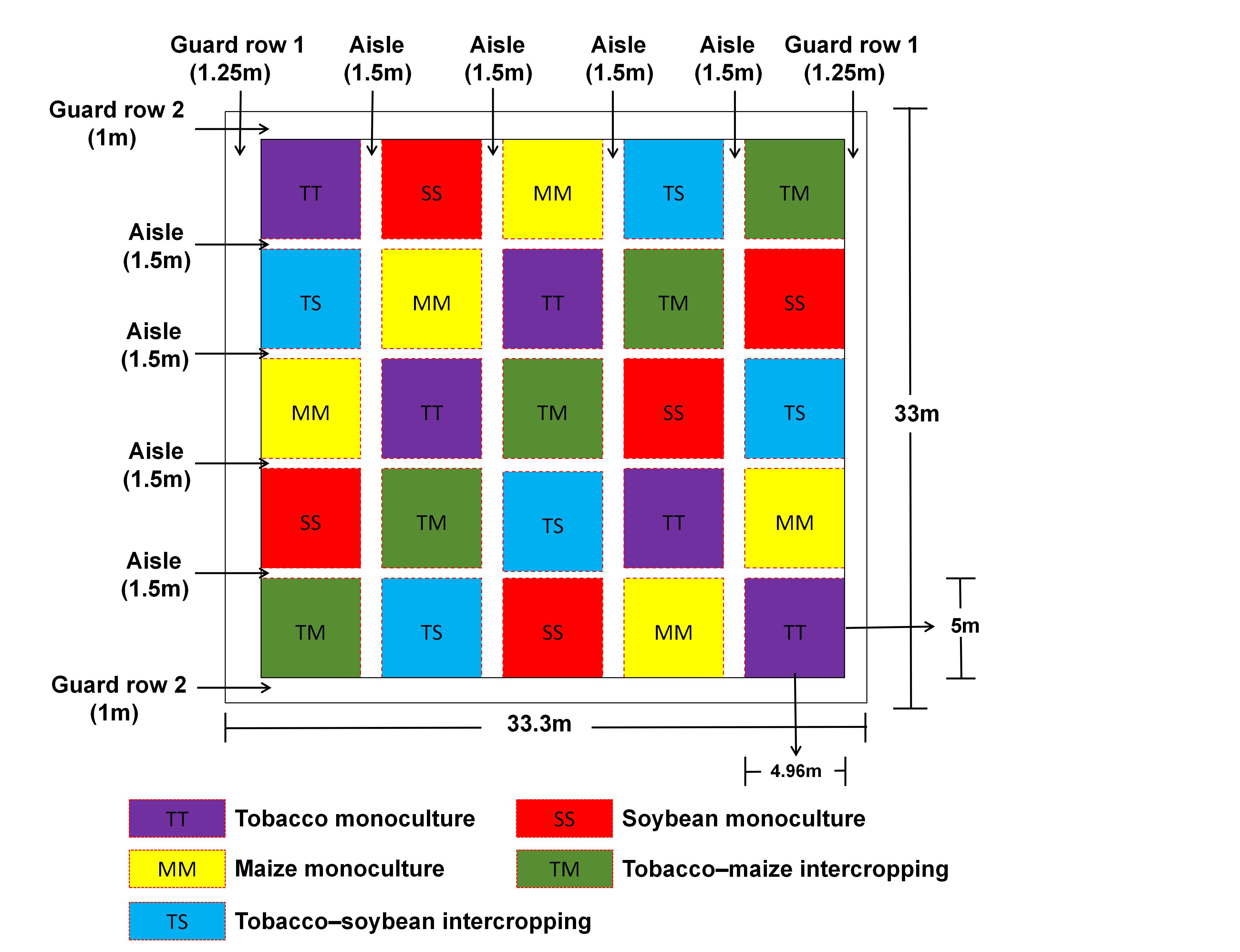


Figure S2. Field layout and design diagram


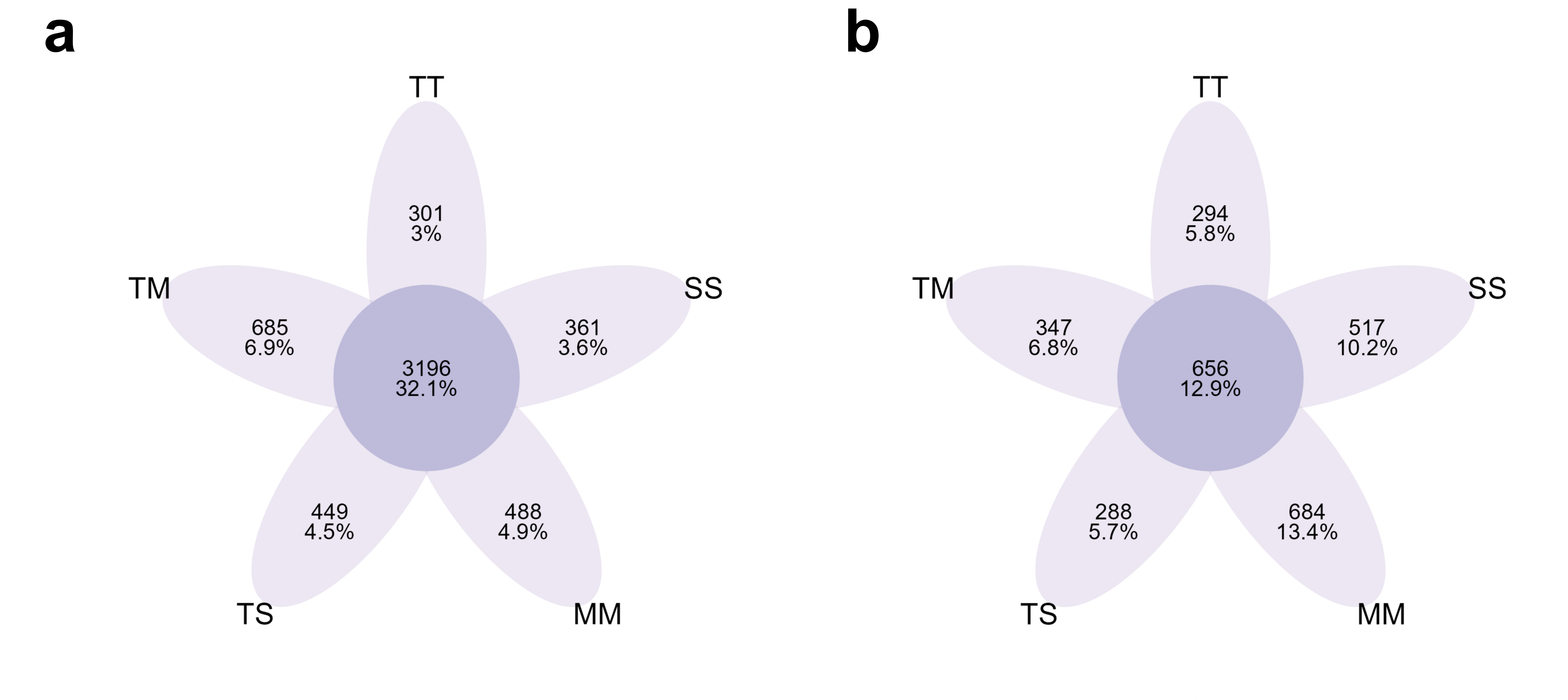


Figure S3. Venn Diagram of Soil Bacterial and Fungal OTUs in Tobacco Intercropping Systems


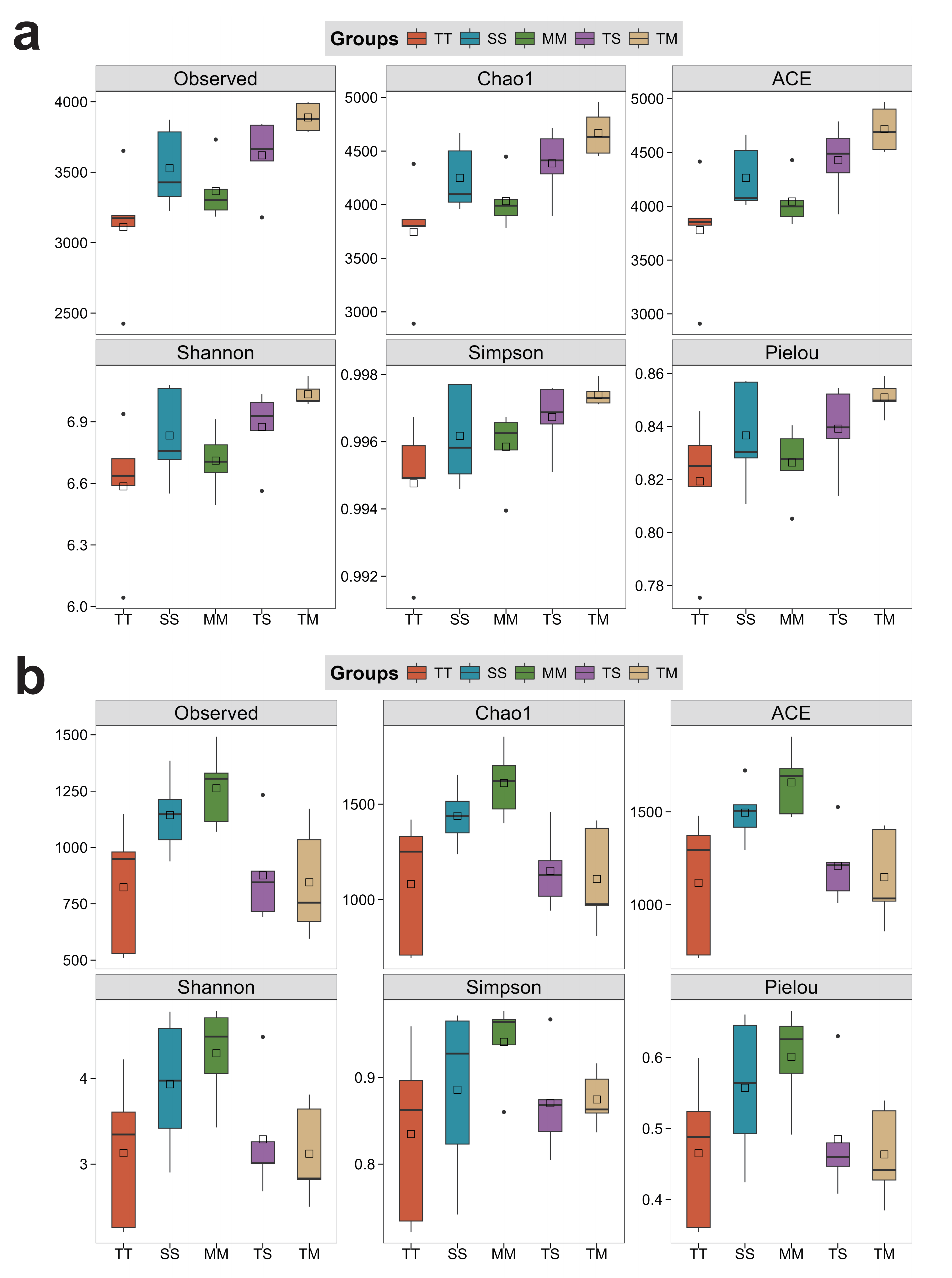


Figure S3. Box Plot of Rhizosphere Microbial Diversity in Tobacco Intercropping Systems

Table S1. Topological Properties of Microbial Networks Across Different Treatments

| Network Parameters | Bacterial | | | | | Fungal | | | | |
| --- | --- | --- | --- | --- | --- | --- | --- | --- | --- | --- |
|  | TT | SS | MM | TS | TM | TT | SS | MM | TS | TM |
| Vertex | 339 | 393 | 404 | 439 | 625 | 84 | 141 | 218 | 111 | 102 |
| Edge | 816 | 1256 | 688 | 991 | 1516 | 78 | 146 | 261 | 118 | 137 |
| Average | 4.81 | 6.39 | 3.41 | 4.51 | 4.85 | 1.86 | 2.07 | 2.39 | 2.13 | 2.69 |
| degree |  |  |  |  |  |  |  |  |  |  |
| Average path length | 1 | 1 | 1 | 1 | 1 | 1 | 1 | 1 | 1 | 1 |
| Network diameter | 1 | 1 | 1 | 1 | 1 | 1 | 1 | 1 | 1 | 1 |
| Clustering coefficient | 1 | 1 | 1 | 1 | 1 | 1 | 1 | 1 | 1 | 1 |
| Density | 0.014 | 0.016 | 0.008 | 0.01 | 0.008 | 0.022 | 0.015 | 0.011 | 0.019 | 0.027 |
| Modularity | 0.948 | 0.924 | 0.979 | 0.969 | 0.979 | 0.95 | 0.945 | 0.964 | 0.942 | 0.923 |

Table S2. Topological Metrics of Integrated Fungal-Bacterial Co-occurrence Networks under Different Cropping Systems

| Network Parameters | Bacterial+Fungal | | | | |
| --- | --- | --- | --- | --- | --- |
|  | TT | SS | MM | TS | TM |
| Vertex | 282 | 333 | 345 | 327 | 307 |
| Edge | 6822 | 10395 | 7937 | 7335 | 5634 |
| Average degree | 48.38 | 62.43 | 44.84 | 44.86 | 36.7 |
|  |  |  |  |  |  |
| Average path length | 2.99 | 2.83 | 3.22 | 3.09 | 3.29 |
| Network diameter | 7 | 7 | 6 | 7 | 6 |
| Clustering coefficient | 0.63 | 0.64 | 0.62 | 0.62 | 0.61 |
| Density | 0.17 | 0.19 | 0.13 | 0.14 | 0.12 |
| Modularity | 6.56 | 9.89 | 3.51 | 6.07 | 0.979 |

Table S2. Mantel Analysis of the Relationships Between Soil Environmental Factors and Microbial Community Structure Under Different Cropping Systems

| Species | Factors | Mantel'r | Mantel'p | Significance |
| --- | --- | --- | --- | --- |
| Bacteria | pH | 0.21 | 0.006 | < 0.01 |
|  | SOM | 0.0262 | 0.365 | ≥0.05 |
|  | TN | 0.0748 | 0.234 | ≥0.05 |
|  | TP | -0.0517 | 0.693 | ≥0.05 |
|  | TK | 0.123 | 0.142 | ≥0.05 |
|  | HN | -0.0753 | 0.87 | ≥0.05 |
|  | AP | 0.027 | 0.336 | ≥0.05 |
|  | AK | 0.0849 | 0.105 | ≥0.05 |
| Fungi | pH | 0.089 | 0.067 | ≥0.05 |
|  | SOM | 0.0284 | 0.318 | ≥0.05 |
|  | TN | 0.00549 | 0.43 | ≥0.05 |
|  | TP | 0.0101 | 0.396 | ≥0.05 |
|  | TK | 0.485 | 0.001 | < 0.01 |
|  | HN | 0.109 | 0.063 | ≥0.05 |
|  | AP | 0.373 | 0.001 | < 0.01 |
|  | AK | 0.494 | 0.001 | < 0.01 |
